# *Lacticaseibacillus rhamnosus* C1 effectively inhibits *Penicillium roqueforti*: Effects of antimycotic culture supernatant on toxin synthesis and corresponding gene expression

**DOI:** 10.3389/fmicb.2022.1076511

**Published:** 2023-01-26

**Authors:** Peipei An, Li Li, Pei Huang, Yin Zheng, Zekun Jin, Sameh A. Korma, Namei Ren, Nan Zhang

**Affiliations:** ^1^Department of Food Science, School of Food Science and Engineering, South China University of Technology, Guangzhou, China; ^2^Innovation and Research Platforms of Life and Health, China-Singapore International Joint Research Institute, Guangzhou, China; ^3^Department of Data Science, School of Software Engineering, South China University of Technology, Guangzhou, China; ^4^Department of Food Science, Faculty of Agriculture, Zagazig University, Zagazig, Sharkia, Egypt

**Keywords:** probiotics, *Penicillium roqueforti*, antifungal mechanism, toxin inhibition, UHPLC-QTOF-MS 2, gene expression

## Abstract

Recently, consumers are increasingly concerned about the contamination of food by molds and the addition of chemical preservatives. As natural and beneficial bacteria, probiotics are a prospective alternative in food conservation because of their antimycotic activities, although the mechanism has not been explained fully at the level of metabolites. This study aimed at investigating the antifungal activities and their mechanisms of five potential probiotic strains (*Lacticaseibacillus rhamnosus* C1, *Lacticaseibacillus casei* M8, *Lactobacillus amylolyticus* L6, *Schleiferilactobacillus harbinensis* M1, and *Limosilactobacillus fermentum* M4) against *Penicillium roqueforti*, the common type of mold growth on the bread. Results showed that C1 emerged the strongest effectiveness at blocking mycelium growth, damaging the morphology of hyphae and microconidia, decreasing DNA content and interfering in the synthesis of the fungal toxins patulin, roquefortine C and PR-toxin, as well as downregulating the expression of key genes associated with the toxin biosynthesis pathways. Further metabonomic investigation revealed that protocatechuic acid with the minimum inhibitory concentration of 0.40 mg/mL, may be most likely responsible for positively correlated with the antimycotic effects of C1. Thus, C1 is expected to be both a potentially greatly efficient and environmental antimycotic for controlling *P. roqueforti* contamination in foods.

## 1. Introduction

*Penicillium roqueforti* commonly colonizes bakery products, meat products and foods required to be preserved at a low temperature because they are rich in nutrients such as proteins, fiber, lipids, and vitamins, causing substantial economic losses and public health concerns (Cisarova et al., 2020). In addition to giving the contaminated foods an unpleasant smell, taste, and appearance, *P. roqueforti* are able to produce a wide array of mycotoxins such as patulin, roquefortine C and PR-toxin (Tannous et al., 2014; Fontaine et al., 2015; Gallo et al., 2015), conferring a human health risk and making the foods unsuitable for consumption. Roquefortine C has been illustrated to carry a neurotoxic effect in mammal and exhibit a suppression of the cytochrome P450 superfamily of hemeprotein enzymes. Patulin may result in acute, subacute and chronic toxicity problems, which was comprised of genotoxicity, immunotoxicity, and neurotoxicity (Li et al., 2021). The PR-toxin, an eremophilane, pertaining to the bicyclic sesquiterpene, possessed nephrotoxicity and abortive toxicity (Hidalgo et al., 2014). Therefore, blocking *P. roqueforti* growth on and mycotoxin contamination of bakery products, meat products and cryogenic foods is currently a major concern.

The main traditional ways of controlling the mold putrefaction of foods have been achieved *via* the supplement of synthetic chemical antiseptic agents (e.g., sorbate or propionate) or treatment with physical preservatives (desiccating, radiation or exposed to low-pressure mercury lamp; Hussain et al., 2021). Nevertheless, the overuse of these preservation techniques has caused the development of fungal resistance, while some of these chemicals cause undesirable biological influence on human safety, so that consumers generally refuse to use these traditional ways to control the growth of microorganisms in foods (Damalas and Eleftherohorinos, 2011; Li et al., 2016). Therefore, the restraint of toxicogenic fungi by biological preservation methods is an emerging and sustainable tactic to meet the increasing market demand for safe antimycotic agents.

Lactic acid bacteria (LAB) have been sorted into “generally recognized as safe,” a label which has become invaluable for the current blossom of the food industry (To et al., 2022). Extensive studies have demonstrated the antimycotic effectiveness of LAB. Treatment with *Pediococcus acidilactici* and *Pediococcus pentosaceus* revealed fungicidal activities against *Fusarium culmorum* and *Fusarium poae* (Juodeikiene et al., 2018). *Lactiplantibacillus plantarum* exhibited fungicidal efficacy against *Aspergillus flavus* and *Aspergillus parasiticus* (Sangmanee and Hongpattarakere, 2014). Furthermore, LAB can yield a wide range of bioactive metabolites *via* fermentation, which may exhibit disparate mycocidal modes. LAB and their antimycotic constituents could substitute physical and synthetic chemical means to achieve the food conservation. Consequently, it is essential to investigate the fungicidal efficacy, mycocide ingredients and action mode of LAB in processed foods.

In an anterior research, we have isolated LAB strains consisting of *Lacticaseibacillus casei* M8 (M8), *Lactobacillus amylolyticus* L6 (L6), *Lacticaseibacillus rhamnosus* C1 (C1), *Schleiferilactobacillus harbinensis* M1 (M1), and *Limosilactobacillus fermentum* M4 (M4) from naturally fermented tofu whey (Fei et al., 2018), which have been shown to possess prebiotic efficacy. *Lacticaseibacillus casei* AST18 presented antifungal activity against *Penicillium chrysogenum* (Li et al., 2014), whereas *Lactobacillus amylolyticus P40 and P50* has been shown to restrict the growth of bacteria (Grosu-Tudor et al., 2014). *Lacticaseibacillus rhamnosus* isolated from cow and goat milk was reported to inhibit *Penicillium commune, P. nordicum* and *P. verrucosum* (Ramos-Pereira et al., 2021), while *S. harbinensis Ca12* was associated with the inhibition of *Candida albicans* (Colares et al., 2021) and *L. fermentum* ATCC 23271 displayed fungicidal effectiveness against *Candida* species (Dos Santos et al., 2021).

There is recently no literature valid which methodically expounds the blocking effects and antimycotic modes of probiotics against *P. roqueforti*. Therefore, in the current study, the suppressive mechanisms toward *P. roqueforti* fungal growth and toxin synthesis of culture supernatants of LAB strains C1, L6, M8, M1, and M4 were investigated for purpose of revealing potentially applications for restraining food corruption by utilizing the culture supernatants of probiotic bacteria. This work provides specific evidence for the development of safer and cost-effective antifungal agents to inhibit *P. roqueforti* contamination of a large number of processed foods.

## 2. Materials and methods

### 2.1. Chemicals and reagents

Analytical-grade anhydrous alcohol, and HPLC-grade acetic acid, acetonitrile and methanol were acquired from Sigma-Aldrich (Shanghai, China).

### 2.2. Microorganisms, culture media, and growth condition

*Lactobacillus amylolyticus* L6 (China General Microbiological Culture Collection Center (CGMCC) No. 9090, NCBI accession number CP020457), *L. rhamnosus* C1 (CGMCC No. 60224, NCBI accession number CP094328), *S. harbinensis* M1 (CGMCC No. 60305, NCBI accession number CP045143), *L. fermentum* M4 (CGMCC No. 62472, NCBI accession number CP089305) and *Lacticaseibacillus casei* M8 (CGMCC No. 62828, NCBI accession number CP094329-CP094331) were previously isolated from spontaneously acidified tofu whey by our lab (Li and Liu, 2015a,b; Fei et al., 2017, 2018), and cultured on the De Man, Rogosa, Sharpe (MRS) medium at 37 ± 0.5°C.

*Penicillium roqueforti* (CGMCC 3.7903) was obtained from the CGMCC (Beijing, China). The test strain was activated and incubated on malt extract agar medium with 0.1 g/L chloramphenicol at 25 ± 0.5°C. The microconidia suspension concentrations were regulated to 1 × 10^7^ microconidia/mL for pursuant experiment.

MRS medium and malt extract medium were acquired from Huankai Microbiology Biotech Inc. (Guangzhou, China).

### 2.3. Antifungal activities and mechanism of culture supernatant of C1, M8, L6, M1, and M4 against mycelial growth and microconidia germination of *Penicillium roqueforti*

#### 2.3.1. Antifungal activities of culture supernatants of C1, M8, L6, M1, and M4 against mycelial growth

The culture supernatants of C1, M8, L6, M1, and M4 were obtained at 4°C through centrifuging at 15,984 g for 10 min. Considering quantification, the supernatant was freeze-dried and used throughout the whole subsequent experiment. Lyophilized powder of culture supernatants of C1, M8, L6, M1, or M4 on MRS at 300 mg/mL were added to malt extract agar medium containing 0.1 g/L chloramphenicol. Aliquots of 8 μl of microconidia suspensions of *P. roqueforti* at 1 × 10^7^ microconidia/mL were inoculated at the core of the malt extract agar medium with 0.1 g/L chloramphenicol. The experimental control was set up using the agar medium without the culture supernatant. Each of the experimental groups and the control groups were carried out as three biological replications. The culture was incubated at 25°C for 7 d and employed to quantify fungal growth. Diameters of the colonies were determinated on the 6th day of incubation in two directions at right-angles to one another. The suppression ratio against mycelial growth was counted in accordance with the formula:

suppression ratio against mycelial growth (MGI %) = [(*d*_c_ – *d*_t_)/*d*_c_] × 100.

where *d*_c_ = average (mm) mycelial growth in control group, *d*_t_ = average (mm) mycelial growth in treatment group (Cisarova et al., 2020).

#### 2.3.2. Antifungal evaluation against microconidia germination

The antifungal effects of the culture supernatants of C1, M8, L6, M1, or M4 on *P. roqueforti* microconidia germination were inspected *via* a light microscope (Hu et al., 2019). Lyophilized powder of five probiotic culture supernatants were used to determinate the inhibition against spore germination. An aliquot (100 μL) of the microconidia suspension was inoculated into 20 mL malt extract medium containing 300 mg/mL of the lyophilized powder of C1, M8, L6, M1, or M4 culture supernatants, then placed at 25°C for 2–6 d to observe the microconidia germination. Then, 150 μl of the above mixture was dropped on a hemocytometer plate and placed under a microscope for observation and counting every 24 h. The number of germinating microconidia out of 200 microconidia in each experimental group and control group was counted. The sample treated with the lyophilized non-incubated MRS medium served as the experimental control, and each treatment was performed as triple biological replicates.

The restraint percentage of microconidia germination was counted in accordance with the following equation: restraint percentage (%) = [(*N*_c_ − *N*_t_)/*N*_c_] × 100% (Qian et al., 2016; Kong Q. et al., 2020). In the formula, N_c_ and N_t_ represent the number of germinating microconidia of the control group and of the experimental group treated with each of the five culture supernatants, respectively.

#### 2.3.3. Scanning electron microscopy (SEM)

Two-day-old *P. roqueforti* exposed to C1, M8, L6, M1, or M4 culture supernatants at 300 mg/mL were observed under SEM referring to the measures described by Li et al. (2020) and Zhang et al. (2016). Approximately 1 × 1 cm fragments were cut from cultures growing on plates. The fragments were washed in 0.1 M phosphate-buffered saline (PBS) at pH 7.2 and fixed using 2.5% glutaraldehyde in 0.1 M PBS for 15 h. The sediments were washed using 0.1 M PBS three times and dehydrated utilizing an ethyl alcohol series (15%–100%). The samples were dryed critically in CO_2_ and sputter coated with gold (IC-50, Shimadzu, Kyoto, Japan). The microstructure features of the conidium and hyphae were examined *via* SEM EVO18 (Zeiss, Oberkochen, Germany) operating at 10.0 kV (Bomfim et al., 2015; Chaudhari et al., 2020).

#### 2.3.4. Detection of *Penicillium roqueforti* DNA by confocal laser scanning microscope (CLSM)

Determination of fungal DNA was implemented *via* CLSM, using DRAQ5, a cell-permeable far-red fluorescent DNA dye, utilized to discern living cells. *P. roqueforti* was treated with C1, M8, L6, M1, and M4 culture supernatants at 300 mg/mL. After adding DRAQ5 (5 mM) to *P. roqueforti* suspensions, the suspensions were complemented with 4 mM EDTA (pH 7.4), mixed homogeneously, and cultivated at 25°C in the dark for 30 min. An aliquot (6 μL) of the stained fungal suspension was pipetted onto a slide and covered with a 20 mm square coverslip. Fungal DNA imaging observation was implemented *via* CLSM (FV1200, Olympus, Tokyo, Japan).

### 2.4. Effect and mechanism of C1, M8, L6, M1, and M4 on *Penicillium roqueforti* toxin production

#### 2.4.1. Detection and quantification of toxins

For the sake of illustrating the restraining properties of the antimycotics against toxin synthesis in *P. roqueforti*, the toxins were extracted in accordance with that of Tannous et al. (2014). Each antimicrobial substance was added to 20 mL malt extract medium (comprising 100 μL 1 × 10^5^ cells/mL of microconidia suspension) to obtain the ultimate antimycotic concentration of 0.06 μL/mL, and the specimen not exposed to culture supernatant served as the control. After 6 d of incubation in the dark at 25°C, the dried mycelia were obtained *via* gathering mycelia and drying them to invariable weight at 40°C. An aliquot (10 mL) malt extract medium was removed from each specimen, then 10 mL methanol/acetonitrile (1:1, v/v) be complemented to extract toxins. The above mixture was oscillated for 2 min *via* the vortex oscillator, the bottom organic layer be then gathered. The extract was dried at 50°C and under vacuum conditions through a vacuum drying oven and dispersed in 1 mL of HPLC-grade methanol. The samples were detected *via* UHPLC-QQQ-MS^2^ assays performed on a 1260-series UHPLC, coupled with an Agilent 6,460 series triple quadrupole mass spectrometer (Bruker-Franzen Analytik GmbH, Bremen, Germany), using a gradient elution comprising water consisting of 0.1% formic acid (A) and methanol (B) with a flow rate of 0.2 mL/min at 22°C; multi-step gradient: 0–5 min, 30% B; 5–10 min, 90% B; 10–13 min, held at 90% B; 13–17 min, 80% B; 17–23 min, 60% B; 23–27 min, 5% B; 27–30 min, 5% B. The negative electrospray ionization (ESI) mode was chosen due to it supplying higher intensity peaks.

#### 2.4.2. Expression level of *Penicillium roqueforti* genes associated with toxin biosynthesis pathway

Mycelium of *P. roqueforti* exposed to the five probiotic culture supernatants at 300 mg/mL was gathered and ground, in accordance with the method described in Subsection 2.5. Extracting RNA and synthesizing DNA were implemented *via* RNAprep Pure Kit and FastKing RT Kit (with gDNase; Sangon Biotech, Shanghai, China), respectively, in accordance with the manufacturer’s protocol. Three genes in the patulin biosynthesis cluster: *patK* (6-methylsalicylic acid synthase), which codes for the first enzyme in the pathway, *patN* (isoepoxydon dehydrogenase, which catalyzes one of the latest steps), and *patL* (transcription factor), were chosen to study the effect of the LAB culture substrates on the biosynthesis of patulin. The expression of genes *rds* (roquefortine cyclo-tryptophan-histidine dehydrogenase) and *rpt* (tryptophan dimethylallyl transferase) related to roquefortine C biosynthesis pathway were tested. We selected four genes from the PR-toxin biosynthesis cluster: *prx1* (short-chain dehydrogenase), *prx2* (aristolochene synthase), *prx3* (quinone oxidase), and *prx4* (alcohol dehydrogenase) to analyze the effect of the LAB culture supernatants on the biosynthesis of PR-toxin. Gene expression was quantified *via* Real-time quantitative polymerase chain reaction (qPCR), SYBR Green (Sangon Biotech, Shanghai, China) monitoring cDNA magnification. The assays were implemented *via* the CFX96 touch system (Bio-Rad, California, USA). Cycling conditions were as follows: activation 3 min at 94°C, 40 cycles for 15 s at 94°C, 15 s at 60°C, 20 s at 72°C and 5 min at 72°C with fluorescence determination. Primer pairs employed in the quantitative analysis were exhibited in Supplementary Table S1 (Hidalgo et al., 2014; Tannous et al., 2014; Kosalkova et al., 2015). *β-tubullin* served as housekeeping gene for standardization to evaluate the changes in the relative expression level of the identified genes. The relative expression level of the identified genes was showed in log2 values as fold changes (Kong Q. J. et al., 2020). Each qPCR assay was reduplicated three times.

### 2.5. Analysis of culture supernatants

#### 2.5.1. Untargeted metabolomics analysis of culture supernatants of C1, M8, L6, M1, and M4

The culture supernatants were obtained at 4°C through centrifuging at 15,984 g for 10 min, and then 20 mL of the supernatant was freeze-dried. An aliquot (400 μL) of methanol/acetonitrile (1:1, v/v) at 4°C were mixed with the sample to extract metabolites. The mixtures were sonicated for 30 min at 4°C and then centrifuged at 15,984 g for 20 min. The supernatants were reserved at 4°C until UHPLC-ESI-MS/MS analysis.

Samples were analyzed on a Waters HSS T3 column (100 × 2.1 mm, 1.8 μm, 120 Å), using a gradient elution comprising water (incorporating 0.1% acetic acid) (A) and acetonitrile (incorporating 0.1% acetic acid) and (B) with a flow rate of 0.2 mL/min at 20°C. The gradient program was as follows: 0.0–2.0 min A/B (90:10 v/v), 6.0–15.0 min A/B (40:60 v/v), 15.01–7.0 min A/B (90:10 v/v).

Data were recorded on a Q Exactive HFX mass spectrometer equipped with the ESI source (Thermo Fisher Scientific, Waltham, USA), utilizing the SIM MS acquisition methods in the Shiyanjia Lab^1^. The ESI source parameters were follows: nebulizing and drying gas at 1.2 Bar, drying temperature at 200°C. The scan modes were set at Auto MS/MS with the mass scan extent of 50–1,500 *m/z*.

#### 2.5.2. Quantitative determination of protocatechuic acid and other crucial components in culture supernatants of C1, M8, L6, M1, and M4

The standard solution of protocatechuic acid, galbanic acid, sclareol, ganoderiol, and solamarine was dissolved in methanol/acetonitrile (1:1, v/v) to prepare the standard solutions at a range of gradient concentrations. These standard stock solutions were analyzed by UHPLC-ESI-MS/MS (Subsection 2.5.1). Standard curves were plotted with peak heights versus standard concentrations. Methods for extraction and analysis of protocatechuic acid and other crucial components in culture supernatants referred to Subsection 2.5.1.

#### 2.5.3. Determination of antifungal effect of protocatechuic acid

The antimycotic efficacy of protocatechuic acid against *P. roqueforti* was assessed *via* the disc diffusion method (Lubbers et al., 2019). 0.1, 0.2, 0.3, 0.4, and 0.5 mg/mL of sterile protocatechuic acid aqueous solution containing DMSO (1%) were used. Aliquots of 100 μL of spore suspensions of *P. roqueforti* at 1 × 10^7^ microconidia/mL was spread homogeneously on the surface of malt extract agar medium with 0.1 g/L chloramphenicol. Disks (6.0 mm diameter) were impregnated with 20 μL of sterile aqueous solution containing DMSO (1%) of protocatechuic acid and placed on the surface of the malt extract agar medium containing *P. roqueforti*, which was incubated at 25 ± 0.5°C for 4 d. The inhibition zones were determined in two directions at right angles to obtain the minimum inhibitory (MIC) and fungicidal concentration (MFC). The minimal concentration inhibiting the visually observable *P. roqueforti* growth was considered as MIC. The MFC was considered the lowest concentration of antimicrobial substances that almost completely blocked *P. roqueforti* growth. Each plate carried a blank disc containing 20 μL of DMSO (1%) as a control.

### 2.6. Data processing and metabolite identification

The raw UHPLC-ESI-MS/MS data were acquired on the Q-Exactive using Xcalibur 4.1 (Thermo Scientific, Waltham, USA), and disposed *via* Progenesis QI (Waters Corporation, Massachusetts, USA). A sequence of processing procedures was implemented, namely peak extraction, peak alignment, baseline correction, de-noising, smoothing, and calibration with default settings. Peak pairs that were monitored in at least 80% of the same-group samples were retained for subsequent analysis. The detected metabolites were analyzed based on fragmentation score (greater than 80%). The reprocessed metabolites were further analyzed, and supervised multivariate models were attained by sPLS-DA *via* R language software. Data was exhibited as mean ± standard deviation (SD) *via* analysis of variance (ANOVA). Pairwise mean comparisons were analyzed *via* Duncan’s multiple range tests, with differences be determined at a 5% level.

## 3. Results and discussion

### 3.1. Inhibition and mechanism against mycelial growth and microconidia germination

#### 3.1.1. Inhibitory effects on mycelial growth

In the present study, the addition of each of the five filter-sterilized probiotic culture supernatant restrained the growth of the test fungus, revealed by the diameter of the fungal colonies. In Table 1, at the probiotic culture supernatant concentration of 300 mg/mL, percentage inhibition by C1, L6, and M8 were 43.17, 27.75 and 23.35%, respectively; the inhibitory effects of M1 and M4 were non-significant and not shown. The extent to which the antimycotics inhibited mycelial growth was ranked in the following order, C1 > M8 > L6 > M1 > M4. Previously reported percentage inhibition of fungal growth of other fungal species by LAB culture filtrates was highly dependent on the probiotic species. The *Candida albicans* was effectively suppressed by S-PT84, a heat-killed preparation of *Lactobacillus pentosus* (Nonaka et al., 2008), whereas the heat-killed Gram-positive LAB *Enterococcus faecalis* exhibited antimicrobial activity on mycelial growth of *Candida* sp. (Roy et al., 2015). However, there are few reports about the inhibition by probiotic bacteria of *P. roqueforti* growth. Therefore, it is important to further evaluate the inhibitory effect of C1, M8, L6, M1 and M4 on *P. roqueforti* growth.

**Table 1:** Inhibition of fungal growth by fermentation supernatant of C1, M8, and L6 at 300 mg/mL.

	Control	L6	M8	C1
Colony diameter (cm)	2.27 ± 0.08	1.74 ± 0.06	1.64 ± 0.12	1.29 ± 0.11
Inhibition rate (%)	-	23.35	27.75	43.17

#### 3.1.2. Antifungal properties against microconidia germination

**Figure 1:**
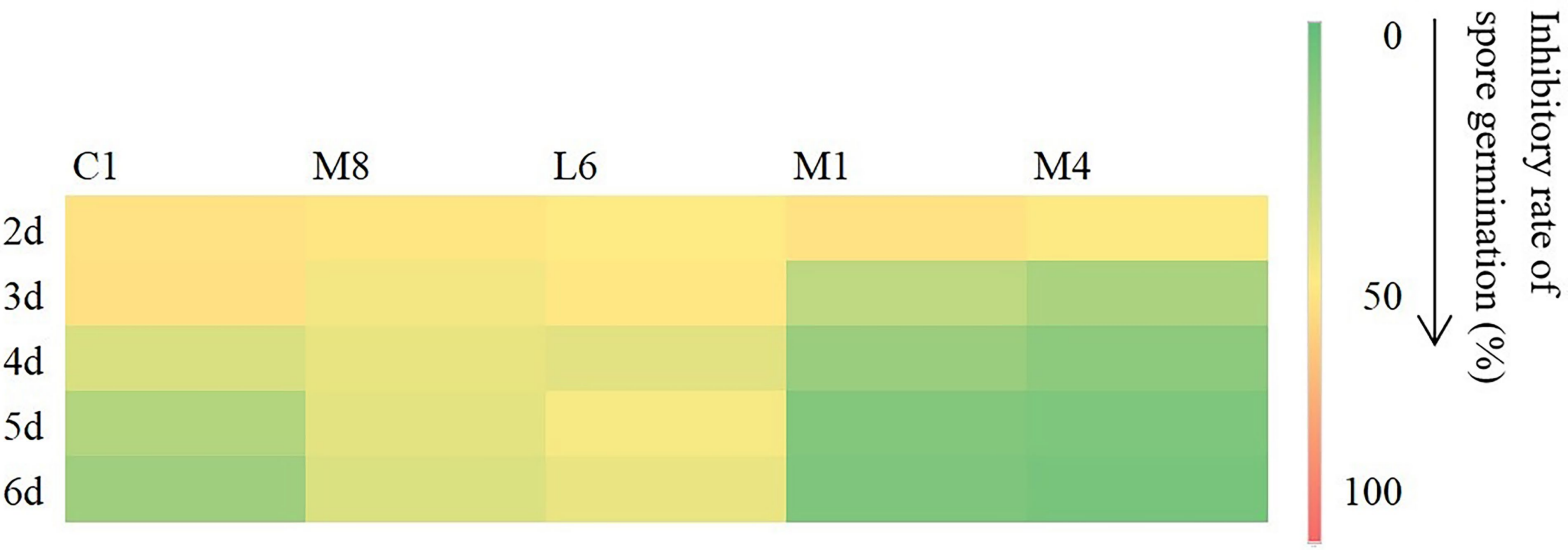
Inhibitory rate (%) of fermentation supernatant of C1, M8, L6, M1, and M4 at 300 mg/mL against spore germination of *Penicillium roqueforti* at different time points after inoculation (2, 3, 4, 5, and 6d). The more green the color, the lower the inhibitory effect, and the more red the color, the higher the inhibitory effect. Values are mean (*n* = 3) ± standard error.

In view of the differences between LAB species of the antifungal effect on mycelial growth of the culture supernatant of C1, M8, L6, M1, and M4, the inhibitory effect against microconidia germination was then measured (Figure 1). Compared with the control, the culture supernatant of probiotic LAB species had a significant inhibitory effect on microconidia germination. The inhibition rates of C1, M8, L6, M1 and M4 against microconidia germination during treatment from 2 to 6 d after exposure were ranked in the following order: C1 (62.43%–25.60%) > M8 (38.51%–33.00%) > L6 (27.79%–8.45%) > M1 (22.29%–3.90%) > M4 (20.66%–3.04%). Although Yadav and co-workers had also demonstrated that *Escherichia coli* BL21 could inhibit microconidia germination of *Aspergillus fumigatus*, *A. flavus*, and *A. niger* (Yadav et al., 2005), little follow-up research on this finding had been carried out. Therefore, the LAB strains C1, M8, L6, M1, and M4 selected in this study are of great significance for their inhibition of *P. roqueforti* microconidia germination.

Based on our results, culture supernatant of three strains of probiotics showed relatively strong antifungal efficacy against both the microconidia and hyphae of *P. roqueforti in vitro*. Furthermore, in view of that the currently used fungicides labeled for use as food preservatives are synthetic chemicals, C1, M8, L6, M1, and M4 act as possible biorational antifungal agents to control fungal growth; in addition, they do not affect the original flavor of preserved foods. Consequently, it is necessary to evaluate the antimicrobial effectiveness of the culture supernatant of C1, M8, L6, M1, and M4 against *P. roqueforti* and to further study the mode of action.

#### 3.1.3. SEM investigation

**Figure 2:**
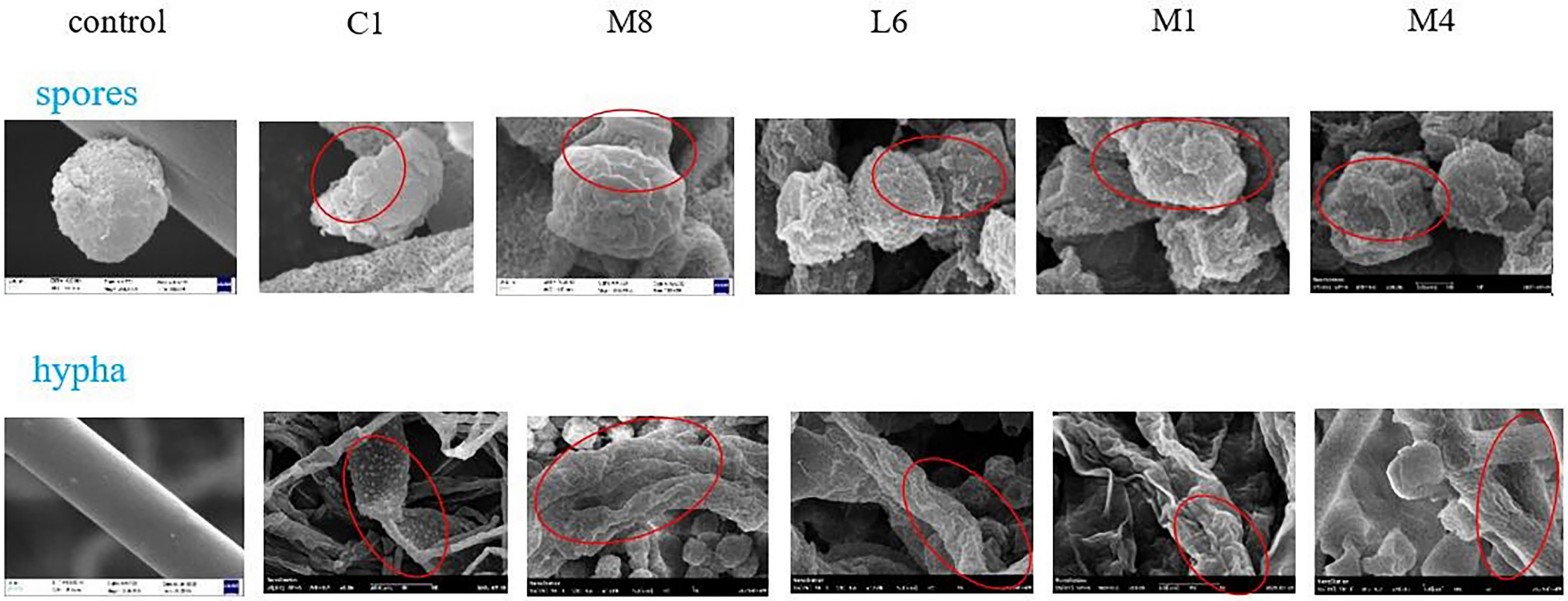
SEM micrographs of hyphae and microconidia of *P. roqueforti* treated by fermentation supernatant of C1, M8, L6, M1, and M4 at 300 mg/mL. Images obtained at 20.0 k × magnification. Red ellipses showed the bending, shrinkage, twisted, broken, or wrinkled hyphae and microconidia.

For the microscopic characteristics of *P. roqueforti* surveyed *via* SEM (Figure 2), the specimens from the control group exhibited integrated structures with uniform hyphae of standardized diameter. When exposed to the culture supernatants of each of the five strains of probiotic LAB tested, varying degrees of effects on the diameter, surface ruggedness, bending or shrinkage were observed for *P. roqueforti* hyphae.

SEM images also showed that microconidia of *P. roqueforti* appeared to be intact and to grow normally in the control group (Figure 2). After treatment with culture supernatants of each of the five strains of probiotics, however, the microconidia appeared wrinkled on account of decreasing cytoplasmic contents, and different percentages of damaged and deformed microconidia were found. Exposure to the culture supernatants of C1, M8, L6, M1, or M4 led to the formation of craters of different sizes on the surface of microconidia. Especially, the microconidia completely sunken under the pressure of C1.

The negative effects of the culture supernatant of five strains of probiotic LAB on microconidia morphology were ranked in the order of C1 > M8 > L6 > M1 > M4. Interestingly, the effect of the probiotic culture supernatant was that both the mycelium and the microconidia exhibited poorly twisted, broken, wrinkled and coarse, accompanied by a flat strip shape, and an apparent cytoplasmic content loss. It has already been reported that the antimycotic effects of probiotic bacteria on many fungi were imputed to bacterial metabolites with low molecular mass and greatly lipophilicity, which could readily destroy cytomembrane and induce cytoplasmic spillage (Chen et al., 2021). Yet, no previous analysis or comparison has been made on the damaging effects of probiotics against fungi from the perspective of microstructure properties, as have been revealed in this research. Therefore, it is significant to note that the destructive effect of C1 > M8 > L6 > M1 > M4 on the morphology of *P. roqueforti* microconidia and hyphae, which is the same as the order for mycelial growth and for microconidia germination.

#### 3.1.4. Effect of the culture supernatants of five strains of probiotics on *Penicillium roqueforti* DNA content

**Figure 3:**
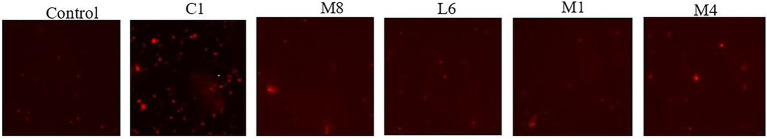
Total DNA content of *P. roqueforti* in the control group and probiotic fermentation supernatant (C1, M8, L6, M1, and M4) treatment group were analyzed by DRAQ5 staining.

In view of the fact that the culture supernatants of LAB strains C1, M8, L6, M1, and M4 had significant damaging effect on the cytoderm and cytomembrane of the fungal hyphae and microconidia, we decided to determine whether the culture supernatants had a negative effect on DNA. The effectiveness of the supernatants on the total DNA content of live fungal cells was inspected through a fluorescent dye DRAQ5 *via* CLSM. Results indicated that DNA content of *P. roqueforti* decreased significantly when exposed to the culture supernatants, with the effect of the supernatants being in the order C1 > M8 > L6 > M1 > M4 (Figure 3).

### 3.2. Effectiveness and mechanism of C1, M8, L6, M1, and M4 culture supernatants in inhibiting *Penicillium roqueforti* toxicity production

A change in microscopic morphology might therefore reflect metabolic changes within fungal cells, which induced us to assume that the antimicrobial substances in culture supernatants might also reduce toxin production.

#### 3.2.1. Effect of probiotic culture supernatant on toxin production

**Figure 4:**
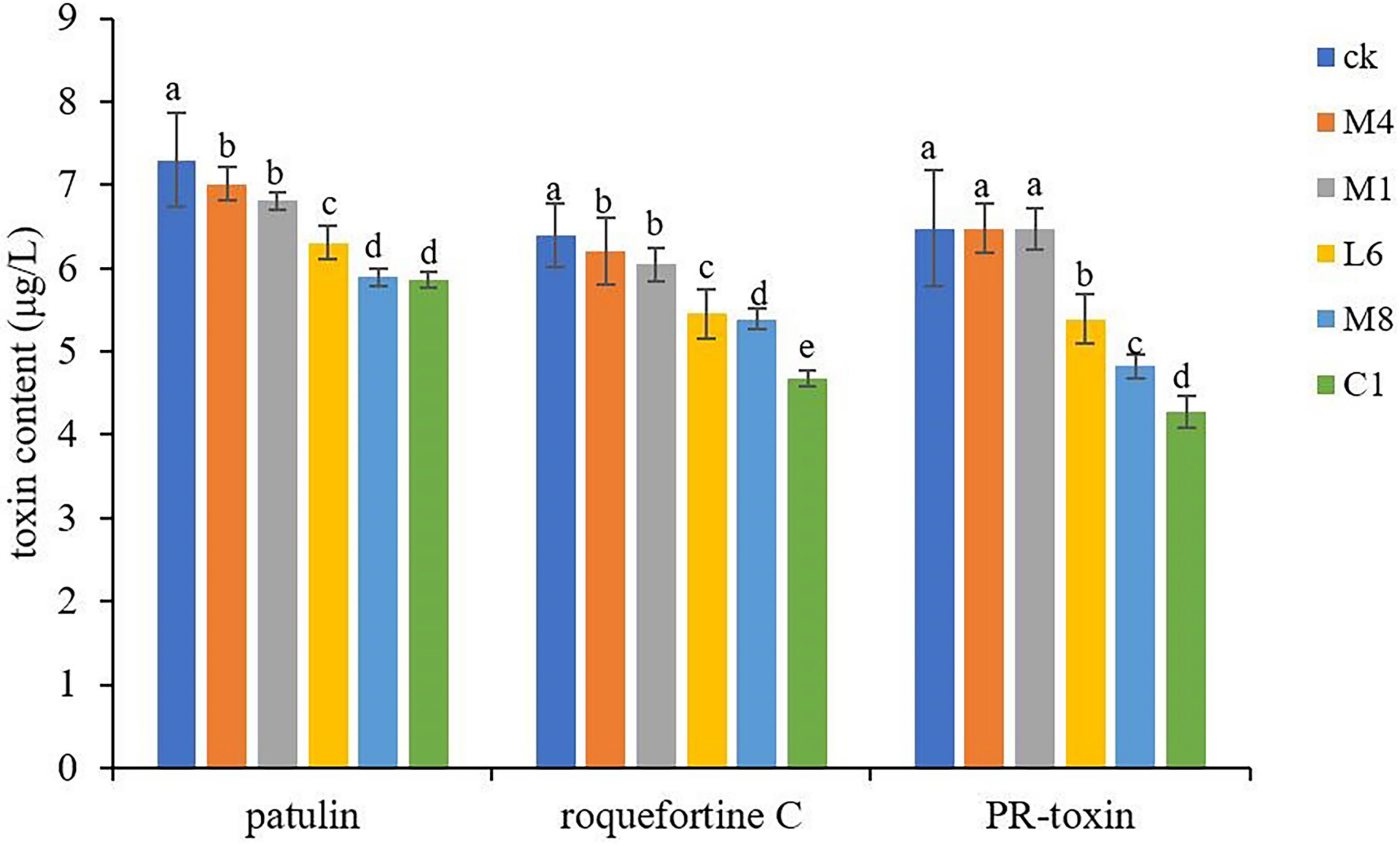
Toxins accumulation in *P. roqueforti* treated with antimycotics at 300 mg/mL. Data are the mean ± SE. The different letters indicate significant (*p* < 0.05) differences according to Duncan’s multiple range test.

The culture supernatant of C1, M8, L6, M1, and M4 generally reduced the biosynthesis of patulin, roquefortine C and PR-toxin (*p* < 0.05), except, for PR-toxin, reduction effectiveness of M1 and M4 supernatant was not significant. Especially, C1 had the most obvious inhibitory effect on toxin production (Figure 4). In the treatment groups, M4, M1, L6, M8, and C1 reduced patulin content production from 7.30 (control) to 7.01, 6.81, 6.31, 5.89 and 5.86 μg/L, respectively, with the inhibition rate ranging from 6.71 to 19.73%; roquefortine C accumulation decreased from 6.40 (control) to 6.20, 6.05, 5.45, 5.39 and 4.67 μg/L, respectively, with an inhibition rate of 5.47% to 11.41%; whereas PR-toxin production decreased from 6.48 (control) to 6.47, 6.47, 5.39, 4.82 and 4.27 μg/L with the inhibition rate of 0.15%–31.64%. Therefore, the inhibitory effect of probiotic supernatant on toxin accumulation was related to the strain of probiotics. The extent of downregulation of toxin concentration in *P. roqueforti* caused by the culture supernatant was ranked in the order of C1 > M8 > L6 > M1 > M4. The only previous studies of microbial inhibition of fungal toxin production showed that the yeast *Saccharomyces cerevisiae* reduced the toxicity of fumonisin B1 and B2 from the phytopathogenic fungus *Fusarium graminearum* (Alassane-Kpembi et al., 2020). The cell-free yeast supernatant after fermentation of kefir grains CIDCA AGK1 exhibited inhibitory activity against *F. graminearum* growth and zearalenone production (Gamba et al., 2016). *Lactobacillus reuteri* CRL 1098 and *Lactobacillus acidophilus* CRL 1014 reduced negative ochratoxin influence on human peripheral blood mononuclear cells (Mechoud et al., 2012).

In the current study, the suppression of hyphal growth and microconidia germination could account for the decrease in toxin production, perhaps as a result of some culture metabolites of C1, L6, and M8 which inhibited toxin biosynthesis.

#### 3.2.2. Genes involved in *Penicillium roqueforti* toxin production are differentially expressed in response to C1, M8, L6, M1, and M4 culture supernatants

**Figure 5:**
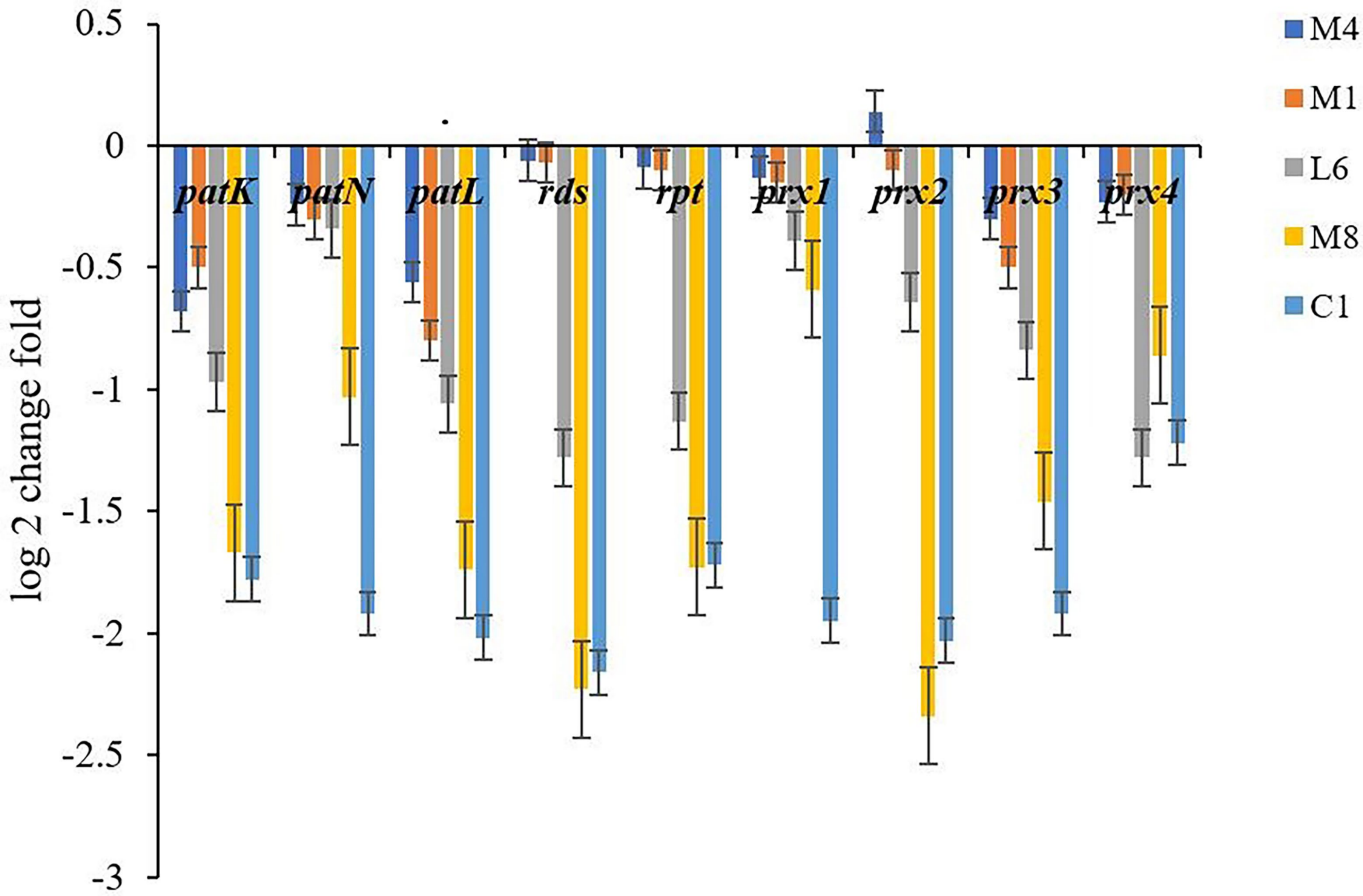
Expression level of genes involved in toxin synthesis in *P. roqueforti* treated with fermentation supernatant of C1, L6, M8, and M4 at 300 mg/mL. Data are the mean ± SE. The different letters indicate significant (*p* < 0.05) differences according to Duncan’s multiple range test.

To further analyze our findings on toxin accumulation, we investigated the key genes expression associated with patulin, roquefortine C and PR-toxin synthesis by qPCR (Figure 5). The *P. roqueforti* treated with the lyophilized non-incubated MRS medium served as the experimental control to reveal the effect of the fermentation supernatant of C1, M8, L6, M1, and M4 on genes expression in *P. roqueforti*. Genes *patK, patN* and *patL* are involved in patulin biosynthesis, *rds* and *rpt* in roquefortine C biosynthesis, and *prx1*, *prx2*, *prx3*, and *prx4* in PR-toxin biosynthesis. Compared with the experimental control, the expression of *patK*, *patN*, *patL*, *rds*, *rpt*, *prx1*, *prx2*, *prx3*, and *prx4* were all significantly downregulated in *P. roqueforti* when exposed to individual culture supernatants from strains C1, M8, L6, M1, and M4. Effects of culture supernatant of the five probiotics on downregulation of gene expression were in the following order: C1 > M8 > L6 > M1 > M4.

Different probiotic culture supernatants had different effects on the corresponding gene expression of different toxins. The results were consistent with the discoveries of the determination of toxin variation. The expression changes of genes caused by different probiotic culture supernatants were attributed to culture metabolites of the strains, with C1 having the strongest downregulation activity on toxin-related gene expression in *P. roqueforti*, which may further reduce the adverse effects of toxigenic molds on food safety and human health.

### 3.3. Analysis of antimicrobial substances produced by the five probiotics

#### 3.3.1. Correlation between antimycotics and antifungal effects of probiotics

**Figure 6:**
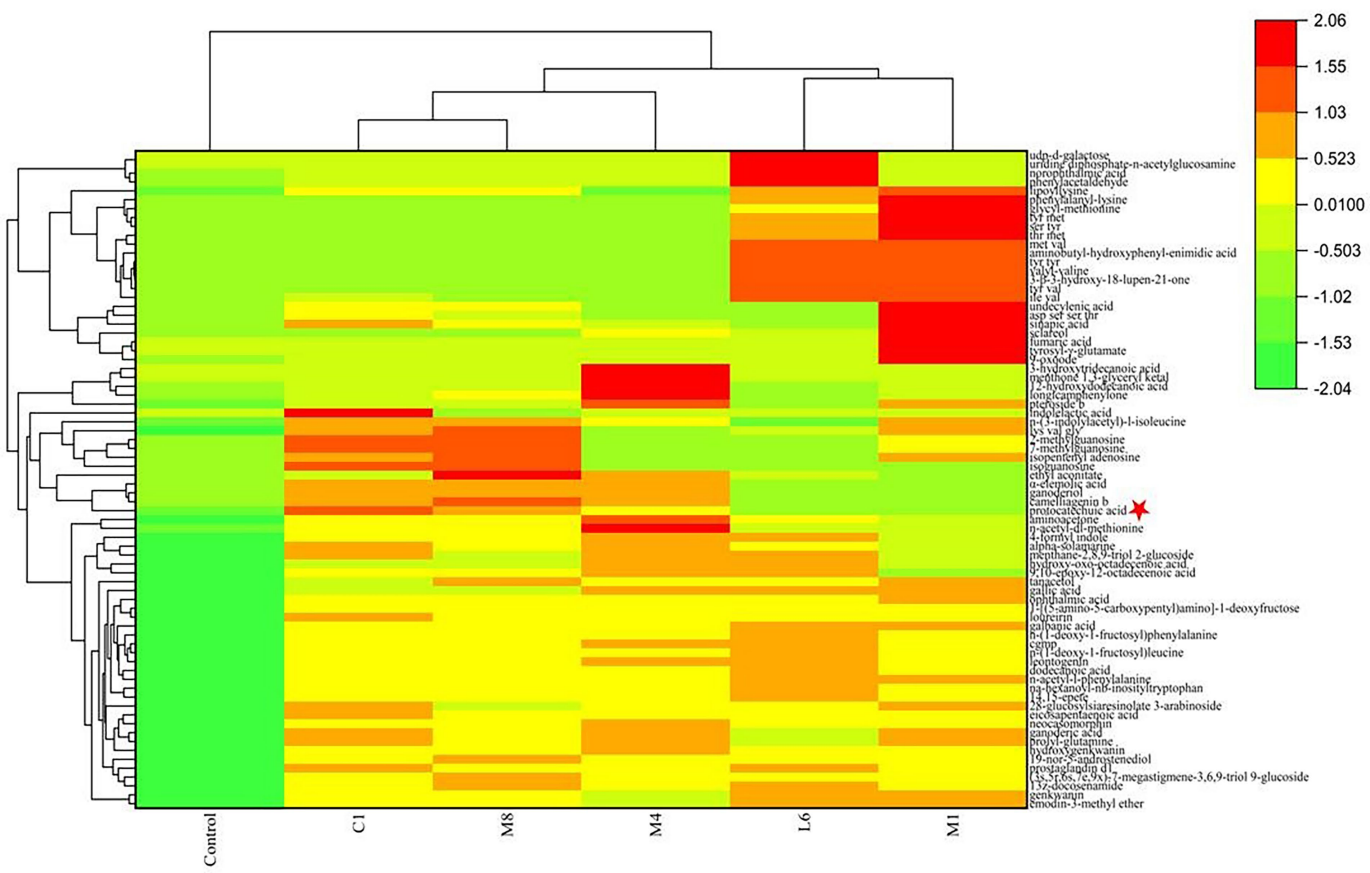
Heatmap reflects the relative abundance of compounds with significant changes in probiotics fermented-MRS compared with the control group.

**Figure 7:**
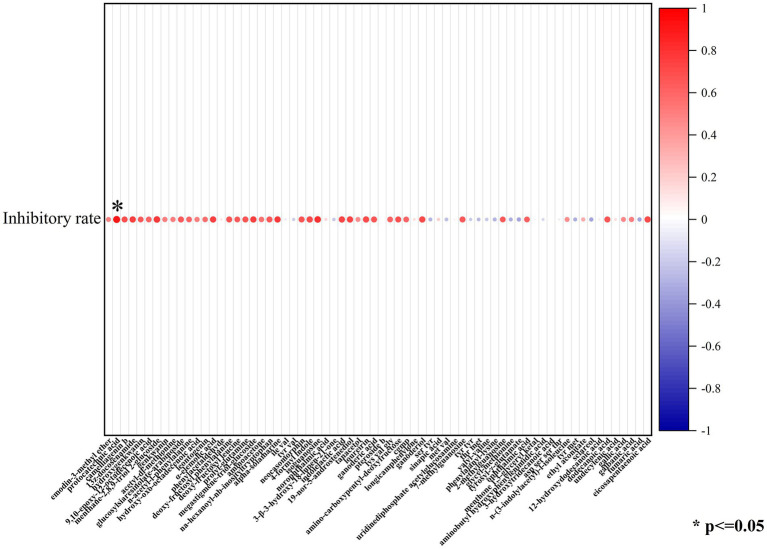
Protocatechuic acid was most responsible for the antimycotic properties of the five probiotics by sPLS-DA.

In order to isolate substances that play a vital role in the antimycotic effect of the five probiotic LAB species, the constituents of lyophilized powder of MRS medium and lyophilized powder of the supernatant after culture of C1, M8, L6, M1, and M4 on MRS were separated by UHPLC-ESI-MS/MS, distinguishing approximately 506 components, 124 of which had fragmentation scores greater than 80%. Among the 124 credible metabolites, the concentrations of 74 substances were accumulated in the culture supernatant group compared with the control group (Figure 6). The increased concentration of 74 metabolites in the C1, M8, L6, M1, and M4 treatment groups compared with the control group and the antifungal efficacy of the five probiotic culture supernatants were analyzed by sPLS-DA using the R language software. It was found that protocatechuic acid was the metabolite most responsible for the antimycotic properties of the five probiotics (Figure 7). Protocatechuic acid, namely 3,4-dihydroxybenzoate, possesses antioxidant, antibacterial and antiaging properties, as well as pharmacological potential in a rat model and clinically for human diseases (Kogure et al., 2021). According to the standard curve, the protocatechuic acid concentrations in the culture supernatants of C1,M8, L6, M1 and M4 strains were determined to be 0.1317, 0.0954, 0.0228, 0.0134 and 0.0854 mg/mL sample, respectively (Table 2). More recently, Myo et al. (2021) reported that the concentration of protocatechuic acid in coffee pulp markedly increased after 24 h of the biotransformation process by *Lactiplantibacillus plantarum* TISTR 543, whereas the total tannin concentration decreased significantly during the fermentation process. In our research, the MRS culture displayed soy ingredients such as isoflavones among the metabolites (Supplementary Table S2). If tannin is also present in MRS medium, it may be converted to gallic acid by esterase, then oxidized by the dehydrogenase and dehydrated by carbonic anhydrase to generate protocatechuic acid (Supplementary Figure S1). Genes encoding most of the enzymes for the above biotransformation processes have been detected in the whole genome of *L. rhamnosus* C1 we measured, such as esterase (EC:3.1.1.1) and carbonic anhydrase (EC:4.2.1.1) (Szwajgier, 2011; Lubbers et al., 2019). Protocatechuic acid may also be converted from tyrosine (Supplementary Figure S2). Tyrosine can be converted to phenylpropanoid and then to 4-4-hydroxycinnamoyl-CoA, and then further converted to hydroxybenzaldehyde and then to hydroxybenzoic acid, finally forming protocatechuic acid (Weber et al., 2012). Tyrosine, hydroxybenzaldehyde and hydroxybenzoic acid were detected among the metabolites of the five probiotics by UHPLC-ESI-MS/MS, but only the gene encoding pyridoxal phosphate-dependent ammonia-lyase, which may catalyze the first reaction, was found in the *L. rhamnosus* C1 genome.

**Table 2:** Contents (mg/mL) of protocatechuic acid and other crucial components in fermentation supernatant of C1, M8, L6, M1, and M4.

	C1	M8	L6	M1	M4
Protocatechuic acid	0.13170 ± 0.00655	0.09540 ± 0.00790	0.02282 ± 0.00227	0.01340 ± 0.00333	0.08540 ± 0.00500
Galbanic acid	0.12980 ± 0.00401	0.15260 ± 0.00340	0.15570 ± 0.00440	0.19200 ± 0.00110	0.19080 ± 0.00673
Sclareol	0.00130 ± 0.00002	0	0.00827 ± 0.00010	0.07740 ± 0.00600	0.03560 ± 0.00900
Ganoderiol	0.12279 ± 0.0017	0.13580 ± 0.00355	0	0	0.15650 ± 0.00870
Solamarine	0.43570 ± 0.00278	0.36500 ± 0.00341	0.36370 ± 0.00405	0.33380 ± 0.00951	0.60440 ± 0.00150

Values were means of 3 replicates ± error value.

Moreover, the antifungal activities of protocatechuic acid against *P. roqueforti* (inhibiting ratio of 23.1, 46.5, 89.0%, 0 and 0), increased with the increasing concentration (0.1, 0.2, 0.3, 0.4, and 0.5 mg/mL). The MIC and MFC of protocatechuic acid were 0.40 and 0.50 mg/mL, respectively, as shown in Table 3. Elansary et al. similarly reported that protocatechuic acid possessed antimycotic efficacy against *Penicillium* species (Elansary et al., 2020). Besides, protocatechuic acid blocked *Aspergillus* species growth and ochratoxin A biosynthesis (Palumbo et al., 2007). The concentration of protocatechuic acid in the MRS supernatant is 0.1317 mg/mL (Table 2), which still inhibits the grow of *P. roqueforti*. Thus, it is possible that protocatechuic acid, together with other substrates such as galbanic acid, sclareol, ganoderiol and solamarine in the supernatant, restrained the mold grow. Further investigation in food matrix should be followed.

**Table 3:** The minimum inhibitory (MIC) and fungicidal concentration (MFC) of protocatechuic acid.

Mold	Protocatechuic acid		MIC (mg/mL)	MFC (mg/mL)
*P. roqueforti*	0.40	0.50

Furthermore, among the 74 substances produced by the five probiotics that increased compared with the control group, in addition to protocatechuic acid, other crucial components that may responsible for antimicrobial effect in culture supernatants were acids (galbanic acid), alcohols (sclareol, ganoderiol) and flavonoids (solamarine; Figure 6). Their contents in the fermentation supernatant of the five probiotics were different (Table 2). Since the contents of galbanic acid, sclareol, ganoderiol, solamarine in C1 were not the highest, their contribution to the fungicidal activity was not as high as that of protocatechuic acid. Organic acid molecules break down the intracellular barrier and cause the fluctuating concentration of metabolites inside and outside the cell (Guo C. X. et al., 2022; Guo Y. J. et al., 2022). Galbanic acid induce beneficial fungicidal activity as a result of exhibiting stronger antioxidant activities and greater bioavailability than the conjugated ester form (Yuan et al., 2019; Ska et al., 2021). The hydrophobic tails of alcohols enter into the hydrophobic core of the lipid bilayer of biological membranes, and subsequently, they disturb hydrophobic interactions among the lipid molecules, bringing about a lessened lipid order an incremental membrane liquidity, which triggers the mycocidal properties (Kleinwachter et al., 2021). Sclareol and ganoderiol with higher radical scavenging activities and pro-oxidant activities are intermediates that confer the oxidative action of H_2_O_2_ (Brudzynski et al., 2012). Sclareol or ganoderiol/H_2_O_2_-induced oxidative stress generated the DNA-damaging activities, which explained the DNA damage and downregulation of toxin-related genes in *P. roqueforti* caused by probiotic culture supernatants. Flavonoids that contain hydroxyl moieties could suppress *P. roqueforti*, by inducing leakage of the microorganism’s nucleic acid and affecting the transcription and metabolism pathways (Ozcelik et al., 2011; Lin et al., 2020). These would explain why the above galbanic acid, sclareol, ganoderiol, solamarine, with potential antimycotic efficacy, may have the positive correlation with the mycocidal properties of the culture supernatant of the five probiotics. It is vital to analyze how the antimycotics were synthesized by the five probiotics and to identify their chemical structures for studying the antimycotic mechanism against *P. roqueforti*. Further studies in are warranted to explore in greater detail the antifungal mechanism of key antimycotic components which inhibit *P. roqueforti* growth and toxin accumulation.

## 4. Conclusion

We investigated the antimycotic activity of C1, M8, L6, M1, and M4 against *P. roqueforti* from the perspective of mycelial growth, microconidia germination, morphological structure, DNA content, toxin production and toxin-related gene expression. C1 exhibited the greatest antifungal effects. From the molecular point of view, the expression alteration of genes related to toxins production was positively correlated with the varying in relevant toxin concentrations, which partly reflected the metabolic variation in *P. roqueforti* treated with the fermentation supernatants of C1, M8, L6, M1, and M4. Lastly, protocatechuic acid with the MFC of 0.50 mg/mL, appeared to be the probiotic metabolite most responsible for the antimycotic properties. Besides, galbanic acid, sclareol, ganoderiol and solamarine were related to the antimicrobial effects. Our research demonstrated that C1 possessed the greatest potential to be used as an alternative conserving agent to block fungal growth effectively.

## Data availability statement

The original contributions presented in the study are included in the article/supplementary material, further inquiries can be directed to the corresponding author.

## Author contributions

PA: conceptualization, methodology, investigation, and writing - review and editing. LL: resources, supervision, and writing-review and editing. PH: software. YZ, ZJ, SK, NR, and NZ: validation. All authors contributed to the article and approved the submitted version.

## Funding

This work was supported by the Key-Area Research and Development Program of Guangdong Province (grant number 2019B020209001).

## Conflict of interest

The authors declare that the research was conducted in the absence of any commercial or financial relationships that could be construed as a potential conflict of interest.

## Publisher’s note

All claims expressed in this article are solely those of the authors and do not necessarily represent those of their affiliated organizations, or those of the publisher, the editors and the reviewers. Any product that may be evaluated in this article, or claim that may be made by its manufacturer, is not guaranteed or endorsed by the publisher.

## Supplementary material

The Supplementary material for this article can be found online at: https://www.frontiersin.org/articles/10.3389/fmicb.2022.1076511/full#supplementary-material

## Footnotes

^1^ www.shiyanjia.com
