# Supplementary material for: Lacticaseibacillus rhamnosus C1 effectively inhibits Penicillium roqueforti: Effects of antimycotic culture supernatant on toxin synthesis and corresponding gene expression
Source: Front Microbiol. 2023 Jan 26;13:1076511. doi: 10.3389/fmicb.2022.1076511 (PMC9909597; doi:10.3389/fmicb.2022.1076511)
Supplement: Supplementary file 1 [file Table_1.docx]

Table S1. Primers for toxins biosynthesis pathway.

| Primers | Nucleotide sequences (5′-3′) |
| --- | --- |
| *β-tubullin-F*  *β-tubullin-R*  *patK-F*  *patK-R*  *patN-F*  *patN-R*  *patL-F*  *patL-R*  *rds-F*  *rds-R*  *rpt-F*  *rpt-R*  *prx1-F*  *prx*1*-R*  *prx2-F*  *prx2-R*  *prx3-F*  *prx3-R*  *prx4-F*  *prx4-R* | TCCAAGGTTTCCAGATCACC  GAACTCCTCACGGATCTTGG  ACTCCTGGTACTGAGTACAGTGAATATGAA  CTCTGGAATCTCACCCACTGC  CTAGCAAAGATTCCCTCGTCAAGT  ATATGCATCGGCGAGGCATT  ACTGGTGTAGGCTCAATCGTGTC  ATTTCTTAATCGTTGCATTTCTCGG  TTCGTTGCAAATACGCTG GCTCAGAACGCAGTC  CACAGTGGCCACCACACTGCACTCGGCGGGGCC  GAATATCTGTGTCTCTTCGCACATATCATAATC  TTGATGCAGCTCCATGTCGATGGTGGCAGACCC  CCATGCCATGGGCCAGAGGCGAAGACAGTG  CCATGCCATGGTTACCAAGTGCAGCCGTTATG  CCATGCCATGGCGGACCGAACCAAGCCAGGAAGA  CCATGCCATGGGACTCCTCCGCCAGAACCT  CCATGCCATGGAGCTGTCTCCGATTTCCTACTCCA  CCATGCCATGGAATGCGCAACAGAACTCCGACTT  CCATGCCATGGGACATTGCCGTGGCCAAC  GTCAAAATTGTGCGCCCGTTCC |

| Metabolite  Table S2. List of metabolites identified based on untargeted metabolomics analysis. | Control | C1 | M8 | L6 | M4 | M1 |
| --- | --- | --- | --- | --- | --- | --- |
| calcium pantothenate | 0 | 886729.5386 | 1454192.866 | 1614202.607 | 1189007.412 | 1510210.259 |
| emodin-3-methyl ether/physcion | 0 | 445224.5149 | 421237.3961 | 546581.3453 | 348682.629 | 601946.1999 |
| l-histidine | 0 | 2326264.548 | 2140775.187 | 3091961.109 | 1882737.515 | 2182092.304 |
| trp phe | 106695.2466 | 4332.946131 | 2899.585998 | 51774.86527 | 1846.659676 | 36998.42068 |
| genistein | 153560.7537 | 2141687.745 | 1774538.379 | 1725366.289 | 1386753.933 | 2064184.691 |
| protocatechuic acid | 0 | 179331.6461 | 125004.7393 | 33370.98857 | 94273.11635 | 16459.50443 |
| acetyl-dl-leucine | 160121.1976 | 127683.1656 | 202147.4122 | 135158.4322 | 180829.7259 | 136056.4937 |
| daidzin | 252822.268 | 100714.2553 | 102737.2411 | 96773.2875 | 99159.31883 | 25024.18835 |
| guanine | 903022.6812 | 496170.3358 | 317271.1004 | 12020170.52 | 864965.8218 | 1986230.47 |
| n-lactoyl-phenylalanine | 112567.8948 | 77887.94115 | 147468.2808 | 61622.86281 | 73825.75134 | 171719.0769 |
| d-proline | 0 | 8079962.378 | 8790627.194 | 11588048.92 | 7528950.455 | 10484226.17 |
| xanthosine | 479152.5905 | 788239.5046 | 603187.6218 | 439715.6151 | 0 | 7920.331043 |
| l-valine | 0 | 42828274.77 | 43862470.34 | 48290943.19 | 43904028.81 | 41882689.88 |
| l-lysine | 147882.3833 | 11242553.98 | 11402155.06 | 16412545.75 | 11465735.4 | 11928586.55 |
| camelliagenin b | 0 | 17650.47504 | 21046.63138 | 0 | 18755.84035 | 0 |
| 13z-docosenamide | 0 | 1271531.724 | 1356949.599 | 1328334.416 | 1122441.485 | 1103140.208 |
| daidzein | 955077.9428 | 1022964.806 | 884232.1844 | 1026764.673 | 754665.5332 | 1580329.745 |
| pro pro leu | 195190.0121 | 33461.60693 | 30142.51978 | 28017.82682 | 35169.52375 | 36844.62823 |
| pantothenic acid | 990089.7458 | 431389.9924 | 686117.5788 | 787120.2031 | 610164.4375 | 720506.1493 |
| hydroxygenkwanin | 0 | 25000.87341 | 25300.82427 | 25150.33479 | 30117.20163 | 25399.36341 |
| 9-oxoode | 0 | 968.1015387 | 958.9698267 | 1152.272317 | 2122.904353 | 12321.24803 |
| soyasaponin i | 274170.5685 | 1249.866436 | 111.8732165 | 552587.2012 | 0 | 500506.1782 |
| 9,10-epoxy-12-octadecenoic acid | 0 | 97424.65634 | 109299.6175 | 150814.903 | 148236.4809 | 64222.2144 |
| (1s,2r,4r,8s)-p-menthane-2,8,9-triol 2-glucoside | 0 | 255393.3759 | 189297.5625 | 252647.8334 | 265169.7581 | 171220.4092 |
| genkwanin | 0 | 443682.5592 | 385294.1138 | 509555.7318 | 337981.2959 | 564407.3517 |
| 14,15-epete | 0 | 21512.69314 | 18112.64752 | 24660.01955 | 20235.95344 | 18211.24629 |
| l-phenylalanine | 115657.0419 | 30225466.25 | 28995088.77 | 34892058.34 | 30932531.1 | 28957462.01 |
| n-acetyl-dl-methionine | 0 | 37840.87563 | 49350.54226 | 30999.45944 | 76036.16911 | 27196.04441 |
| 13,14-dihydro pgf-1a | 0 | 89505.81584 | 52814.36802 | 49831.79963 | 51440.6637 | 80439.73585 |
| 28-glucosylsiaresinolate 3-arabinoside | 0 | 104164.3253 | 69533.57422 | 95752.21127 | 96265.61518 | 100255.2285 |
| salicylic acid | 211629.3943 | 44347.94919 | 40679.83974 | 54460.69569 | 33907.65784 | 42319.79635 |
| n-acetyl-l-phenylalanine | 0 | 80696.74087 | 86067.45131 | 92468.34833 | 79952.60565 | 91633.61419 |
| ((+-))-(e)-13-hydroxy-10-oxo-11-octadecenoic acid | 0 | 7830.669707 | 7143.672885 | 12617.165 | 12717.21468 | 6958.494134 |
| leontogenin | 0 | 68844.5817 | 71893.1495 | 83734.35092 | 84642.82401 | 78148.32168 |
| genistin | 869314.6697 | 34264.68812 | 93639.10349 | 46896.27634 | 156185.264 | 12518.1486 |
| azelaic acid | 23124.31943 | 22064.94534 | 21420.5166 | 18984.36017 | 16220.65699 | 17525.30647 |
| trp asp | 341656.4655 | 3044.567101 | 559.1645431 | 106518.5004 | 1559.978068 | 105796.8635 |
| α-elemolic acid | 0 | 54315.30634 | 54574.86954 | 0 | 48881.75653 | 0 |
| prostaglandin d1 | 0 | 44829.78959 | 39807.8285 | 41575.48657 | 34658.71469 | 35190.7242 |
| phenylacetaldehyde | 83956.2044 | 1889239.018 | 1765226.682 | 26477425.74 | 2291001.628 | 2719611.749 |
| adenosine | 65219940.12 | 476313.4134 | 466240.262 | 15341.61309 | 0 | 0 |
| n-(1-deoxy-1-fructosyl)phenylalanine | 0 | 38848913.45 | 37318187.91 | 41934889.46 | 39399831.81 | 39872086.02 |
| n-(1-deoxy-1-fructosyl)leucine | 0 | 73163500 | 74189894.35 | 81697650.4 | 80028229.08 | 77030099.63 |
| glu phe | 762433.4579 | 3235.069889 | 3423.005749 | 190795.7099 | 3581.028726 | 69532.4527 |
| prolyl-glutamine | 0 | 27587.38852 | 22162.27215 | 20397.43565 | 27172.80024 | 26995.09503 |
| ferulic acid | 28736.82885 | 3690.810177 | 19631.19183 | 1167.982948 | 820.3262797 | 28736.82885 |
| phe asp ile | 363722.6034 | 137140.8001 | 153993.8203 | 107491.037 | 110274.7074 | 159889.8375 |
| asn phe | 2194790.783 | 69172.70803 | 41955.0249 | 562264.0533 | 47773.19745 | 1407164.379 |
| asp-phe | 1967961.428 | 91233.53163 | 83921.35236 | 127183.1436 | 89246.02816 | 138239.3763 |
| (3s,5r,6s,7e,9x)-7-megastigmene-3,6,9-triol 9-glucoside | 0 | 649731.0768 | 715483.6431 | 623748.793 | 539744.2994 | 576371.3926 |
| aminoacetone | 0 | 242014.3189 | 273744.0836 | 263499.9468 | 399636.1954 | 236744.2603 |
| na-hexanoyl-nb-inosityltryptophan | 0 | 149277.175 | 129425.77 | 177471.3284 | 153353.9278 | 146269.485 |
| alpha-solamarine | 0 | 593100.5611 | 478128.0022 | 531965.9961 | 667506.2453 | 409347.4905 |
| threoninyl-phenylalanine | 29231.33725 | 25637.69763 | 5293.143893 | 1679282.971 | 6131.556742 | 1569938.41 |
| d-tryptophan | 3398911.928 | 17977.58867 | 27092.82883 | 40137.96325 | 27416.92825 | 45259.93544 |
| l-glutamic acid | 3297503.962 | 3961286.426 | 3834898.349 | 4723937.774 | 3676249.747 | 5530527.789 |
| ile val | 0 | 2218419.075 | 196245.5258 | 12281339.64 | 175932.3289 | 10627229.91 |
| tyr val | 0 | 46760.21726 | 40583.34076 | 1510567.35 | 59290.69372 | 1407503.003 |
| thr leu | 3180206.2 | 320203.7361 | 406341.6196 | 5381875.43 | 236740.1462 | 6023781.618 |
| gamma-glutamylphenylalanine | 887973.9117 | 13286.65359 | 3070.457361 | 862926.7315 | 9536.346324 | 1125688.241 |
| n-[[3-hydroxy-2-(2-pentenyl)cyclopentyl]acetyl]isoleucine | 0 | 11799.50951 | 13351.27982 | 6528.294138 | 5345.068129 | 12160.8471 |
| leu thr val | 1017539.016 | 617367.1506 | 870520.3567 | 339331.4356 | 389957.3774 | 826307.5844 |
| gly val val | 3323308.129 | 1040740.837 | 1435145.626 | 1021642.27 | 1260660.93 | 1468454.49 |
| neocasomorphin | 0 | 2229755.246 | 2172201.049 | 1931228.269 | 2328155.453 | 2229758.944 |
| 4-formyl indole | 0 | 2591508.817 | 2916459.7 | 3233682.004 | 3084705.927 | 2026243.96 |
| citric acid | 68884070.8 | 17886539.72 | 17624286.53 | 19939244.68 | 155646.2595 | 940334.4898 |
| asp leu lys | 1116679.101 | 867425.8642 | 1025694.323 | 580446.8423 | 451719.3088 | 1019970.875 |
| isoguanosine | 0 | 962027.3269 | 858348.3574 | 73245.9954 | 0 | 0 |
| norophthalmic acid | 0 | 160956.0943 | 123448.694 | 1265124.784 | 120656.0689 | 129396.6562 |
| 2-isopropylmalic acid | 73893.64087 | 175180.4289 | 181126.7562 | 199851.2457 | 180762.1606 | 188572.0737 |
| 3-β-3-hydroxy-18-lupen-21-one | 0 | 902.6045314 | 0 | 138282.5942 | 0 | 134795.4409 |
| indolelactic acid | 41425.15308 | 226809.719 | 38237.40253 | 40085.5069 | 66736.65529 | 49660.44199 |
| sphinganine | 89443.28339 | 95112.56537 | 82387.14934 | 90937.34628 | 123764.867 | 86952.98511 |
| inosine | 674530.3752 | 1200316.189 | 844150.3734 | 212584.5411 | 0 | 0 |
| 19-nor-5-androstenediol | 0 | 159965.1298 | 186660.2635 | 159736.8681 | 169917.3696 | 145496.0938 |
| tanacetol | 0 | 56296.25057 | 82377.75527 | 79689.16587 | 79471.06203 | 83382.4438 |
| ile leu thr | 332965.1519 | 128896.7323 | 219287.1709 | 210829.7866 | 119797.3977 | 194023.9813 |
| loureirin | 0 | 3462166.999 | 3220141.709 | 3246060.071 | 3043483.584 | 3331098.951 |
| ganoderic acid | 0 | 78833.15418 | 69163.02478 | 59513.18997 | 80928.62739 | 79150.80931 |
| ser leu ile | 2000445.36 | 568845.4983 | 937857.7897 | 485139.8433 | 621728.964 | 862304.3462 |
| pteroside b | 0 | 231682.6208 | 321113.7719 | 193065.0867 | 919941.73 | 697987.2436 |
| ser leu | 6722730.594 | 200721.0818 | 151070.2213 | 2548830.076 | 147908.5385 | 4828963.725 |
| lys val gly | 0 | 627197.2147 | 746903.407 | 400785.7 | 266970.6313 | 667940.0309 |
| 1-[(5-amino-5-carboxypentyl)amino]-1-deoxyfructose | 0 | 9637434.112 | 8825620.351 | 9382246.873 | 8687779.951 | 9531503.519 |
| acetyl-l-tyrosine | 198835.342 | 46502.9425 | 42885.72263 | 53831.52843 | 46590.26851 | 41093.91555 |
| cgmp | 0 | 1723238.616 | 1827421.056 | 1995972.654 | 2014181.313 | 1888908.627 |
| abscisic alcohol 11-glucoside | 0 | 790529.6455 | 785548.7173 | 690466.941 | 861279.198 | 843619.6912 |
| 12,13-epoxy-11-hydroxy-9,15-octadecadienoic acid | 0 | 320397.1666 | 347746.7931 | 335036.5661 | 339280.9351 | 373645.8593 |
| 6-beta-hydroxymedroxyprogesterone | 0 | 12689.73403 | 14364.60538 | 17225.23969 | 14322.53205 | 17201.11281 |
| longicamphenylone | 0 | 1129.12751 | 2113.505277 | 0 | 8633.499224 | 394.2530128 |
| 3,20,27-trihydroxy-1-oxowitha-5,24-dienolide 3-glucoside | 0 | 4265840.104 | 4416505.538 | 4862588.288 | 4124840.722 | 4391158.824 |
| 3'-ump | 0 | 3020020.733 | 3506006.095 | 4557546.217 | 3720069.272 | 4973359.799 |
| hypoxanthine | 510838.528 | 375293.1863 | 488256.4903 | 8817791.787 | 3096204.042 | 3826520.657 |
| fluvastatin | 0 | 27514.83577 | 22085.51513 | 30525.35028 | 20770.88071 | 27896.52201 |
| gly pro ile gly | 1227488.006 | 837814.1631 | 1073584.511 | 1176293.248 | 1047368.974 | 1194039.882 |
| arginyl-proline | 0 | 4805366.762 | 4703382.739 | 4650735.876 | 5030328.898 | 4945799.217 |
| mannitol | 0 | 311646.4697 | 607195.1619 | 252781.7026 | 2584377.039 | 216706.6767 |
| ile ile thr | 0 | 154002.5512 | 232976.0334 | 231910.8021 | 75541.37278 | 205168.9171 |
| ser lys | 0 | 708797.12 | 687637.9791 | 585884.6631 | 417865.954 | 871098.6295 |
| l-tyrosine | 6620527.867 | 22412.21266 | 23233.81971 | 26456.56457 | 20090.43675 | 23674.66548 |
| glycylprolylhydroxyproline | 91287.2666 | 428203.6346 | 346215.0702 | 476721.0135 | 385295.2076 | 425801.0769 |
| adenine | 1695429.231 | 683311.4151 | 698085.4497 | 61521.46703 | 25872.45053 | 2081.215708 |
| asp ile | 2117097.314 | 2336812.773 | 2264338.039 | 3694922.806 | 2121140.464 | 4319744.078 |
| furanofukinin | 0 | 118068.2949 | 126961.7511 | 124106.9327 | 122310.3062 | 110416.5762 |
| corchorifatty acid | 0 | 53924.15214 | 52447.21299 | 56419.67938 | 46044.60813 | 50993.88699 |
| val ile gly | 0 | 164348.6052 | 201589.2941 | 326953.7452 | 155649.0881 | 243239.4156 |
| diprotin | 1863369.46 | 1192875.285 | 1519115.758 | 630914.4303 | 932364.7415 | 1633396.919 |
| asp phe leu | 0 | 50649.04309 | 43590.79245 | 53372.9724 | 41708.02615 | 49977.38756 |
| 12,13-epome | 0 | 4954.603812 | 2054.516739 | 3677.921656 | 8481.174781 | 25785.2905 |
| d-myo-inositol-3,4,5-triphosphate | 0 | 263850.8905 | 259153.2517 | 326640.9364 | 290765.8914 | 281742.3542 |
| 2,5-dimethylbenzaldehyde | 6303.218793 | 14504.26736 | 9527.701582 | 19087.04221 | 19299.8086 | 18217.30805 |
| l-β-aspartyl-l-leucine | 3757307.711 | 1473388.943 | 1535572.573 | 1650654.679 | 1436363.818 | 2178080.579 |
| 5-hydroxytryptophan | 151413.8712 | 1278808.558 | 1373041.025 | 1738546.526 | 1224588.343 | 1287429.519 |
| glutamyltyrosine | 338153.1418 | 905940.2151 | 906982.5256 | 916780.1429 | 869283.2577 | 0 |
| 9,10-dihydroxy-8-oxo-12-octadecenoic acid | 0 | 217844.2409 | 195192.6567 | 227738.616 | 193517.9782 | 213837.2246 |
| isoleucyl-glutamine | 69282.58696 | 7825.608022 | 0 | 47741.41453 | 4577.870168 | 0 |
| n-acetyltyramine | 29049.97034 | 126075.6142 | 126986.0428 | 133356.8594 | 110717.3492 | 134444.2766 |
| 4-vinylguaiacol | 0 | 412044.3756 | 413250.855 | 466002.7572 | 441945.9316 | 432446.0284 |
| cis-zeatin-9-n-glucoside | 0 | 2803515.128 | 2886643.473 | 2718779.305 | 2911012.219 | 2766861.416 |
| serylphenylalanine | 0 | 32485.3897 | 7857.917687 | 1243025.164 | 5715.256811 | 1997781.08 |
| n-acetyl-l-alanine | 0 | 1353363.07 | 1268093.544 | 1469329.376 | 1025277.194 | 1355374.521 |
| kynurenine | 65867.89786 | 115223.9049 | 107395.3704 | 123613.2099 | 113482.7354 | 131300.2188 |
| calenduloside methyl ester | 0 | 439373.4131 | 421975.4645 | 446928.3382 | 446322.3336 | 155677.7263 |
| rifampin | 0 | 2442738.619 | 2330000.229 | 3004151.104 | 2531742.969 | 2484315.479 |
| steviobioside | 0 | 205461.6247 | 135393.0066 | 422677.9792 | 177737.749 | 165487.3246 |
| pelargonidin | 205576.2662 | 53534.19048 | 42083.67209 | 44026.70036 | 36467.31061 | 68619.45851 |
| corchoroside | 0 | 123364.498 | 132781.4581 | 169369.5425 | 153834.2529 | 141924.6747 |
| posaconazole | 0 | 676876.9341 | 646429.3176 | 272636.1856 | 652618.4922 | 223791.614 |
| hydroxyhomodestruxin b | 0 | 51702.53293 | 50169.8643 | 44474.72258 | 81966.83757 | 30057.80803 |
| dopamine | 0 | 8226.364081 | 12024.00841 | 47725.28993 | 8828.349231 | 11025.76995 |
| l-malic acid | 0 | 2638416.585 | 1801507.706 | 925597.0891 | 3464438.286 | 194379.7043 |
| ser ser leu | 0 | 111913.396 | 129807.5497 | 70841.68451 | 109331.2531 | 152673.5261 |
| phe glu thr | 0 | 138033.0065 | 206040.5649 | 90254.93841 | 123703.3562 | 200688.5385 |
| n-acetyl-a-neuraminic acid | 0 | 516282.7488 | 490587.4431 | 585370.6804 | 2273462.106 | 1997319.577 |
| pyrophosphate | 0 | 397916.6942 | 432365.2996 | 320607.88 | 498474.7128 | 468159.9635 |
| l-arginine | 0 | 11984491.76 | 11953964.41 | 12938563.43 | 65842.04038 | 11905301.22 |
| ganoderiol | 0 | 167156.4795 | 177949.4207 | 0 | 172851.1671 | 0 |
| cynaratriol | 0 | 24748.7183 | 24022.53545 | 21658.92018 | 25578.18797 | 29465.96289 |
| l-cis-cyclo(aspartylphenylalanyl) | 94793.77658 | 57050.98424 | 48718.08634 | 43105.46611 | 40864.13557 | 47583.36751 |
| (s)-10,16-dihydroxyhexadecanoic acid | 0 | 89282.76111 | 71971.37466 | 66146.64197 | 48588.10376 | 126666.4144 |
| l-isoleucine | 0 | 111034510.3 | 113818967.8 | 133240806.8 | 132649310.9 | 126805749.3 |
| pe(14:1(9z)/14:0) | 0 | 244384.8631 | 219196.2674 | 257892.5816 | 213029.5383 | 229149.8715 |
| adenosine 3'-monophosphate | 1962620.074 | 879869.5864 | 1506452.388 | 5440501.501 | 4228836.522 | 3257419.311 |
| n,n-dimethyl-safingol | 107736.5391 | 298578.2102 | 276628.5511 | 245832.364 | 318883.0739 | 313581.2329 |
| ser tyr | 0 | 19051.19689 | 0 | 304851.4869 | 21136.9774 | 611794.5878 |
| urocanic acid | 116925.5707 | 115964.5199 | 109042.5964 | 115786.8997 | 114003.8767 | 102883.9214 |
| prosopinine | 0 | 32036.11063 | 30607.827 | 30839.87463 | 30199.48638 | 31830.67517 |
| enol-phenylpyruvate | 0 | 48315.45179 | 48924.73575 | 21949.02179 | 16063.10801 | 48956.45407 |
| n-deisopropyl-fluvastatin | 0 | 35595.84282 | 38421.10534 | 566.1703117 | 12161.58289 | 173861.454 |
| 13,14-dihydro pge1 | 0 | 78891.0037 | 107167.2991 | 119152.7179 | 109115.3969 | 65885.48573 |
| piceatannol | 0 | 57595.10357 | 43799.64765 | 45527.20184 | 33438.3439 | 77095.54908 |
| hydroxyprolyl-γ-glutamate | 682540.8864 | 2274725.865 | 2187139 | 2167289.764 | 2204179.961 | 1902077.455 |
| ovalicin | 0 | 174822.6663 | 133885.0828 | 132317.7269 | 192321.7856 | 183199.2812 |
| glutamylproline | 3229385.504 | 2573947.372 | 2553949.716 | 3207395.673 | 2675632.653 | 3079021.849 |
| formyl-5-hydroxykynurenamine | 153143.2448 | 150425.5799 | 168547.9924 | 233489.5037 | 172007.3994 | 212311.9116 |
| pyridine n-oxide glucuronide | 0 | 174706.2901 | 178839.8068 | 171211.2694 | 151311.6049 | 176765.9601 |
| isopentenyl adenosine | 0 | 121614.3031 | 131335.3397 | 0 | 0 | 106177.8448 |
| ile gly val | 0 | 2190413.842 | 3015533.547 | 1226902.571 | 2615271.455 | 2765838.113 |
| c16 sphinganine | 1848911.183 | 1335392.873 | 1241037.56 | 1377728.394 | 1347626.948 | 1172322.416 |
| his leu gln | 0 | 1097103.012 | 1590832.9 | 174589.1509 | 150018.1332 | 1561823.713 |
| agaritinal | 0 | 34512.81251 | 27668.74143 | 30270.79577 | 27693.29706 | 34680 |
| gluconolactone | 0 | 1760007.86 | 2386243.845 | 1874245.151 | 2274654.783 | 2440496.232 |
| thr thr ile | 0 | 39480.67251 | 52168.04817 | 12865.082 | 32552.82267 | 49580.13889 |
| 3-β-hydroxy-22(30)-hopen-29-al | 0 | 2366.784674 | 986.3183346 | 146445.4492 | 918.6095461 | 122205.9142 |
| 3-indolepropionic acid | 14304.87944 | 16817.01952 | 16429.24141 | 17837.05684 | 17907.95136 | 17425.42359 |
| hydroxyfluoroprednisolone butyrate | 0 | 164896.9995 | 192819.2068 | 146309.1541 | 190024.1697 | 156277.2926 |
| l-phenylalanyl-l-hydroxyproline | 0 | 3165.998531 | 4304.628276 | 9197.445232 | 5125.935596 | 72185.85747 |
| glycyl-arginine | 1035871.319 | 376120.8359 | 341383.7552 | 306325.7414 | 52828.54312 | 586922.8528 |
| cytidine monophosphate | 0 | 4032778.202 | 3657015.243 | 3763317.811 | 3739994.095 | 3579237.19 |
| guanidylic acid | 0 | 180633.3134 | 106136.4472 | 126404.4306 | 705526.5743 | 14668.01511 |
| ile asp thr | 0 | 271583.2608 | 272299.7886 | 338658.4006 | 252135.8598 | 317756.2283 |
| asp his leu | 0 | 236527.7891 | 250128.1458 | 301022.3254 | 228438.5456 | 268660.2975 |
| sinapic acid | 0 | 10661.45022 | 8808.592246 | 0 | 3387.295982 | 17750.56581 |
| uric acid | 489184.7797 | 164710.1945 | 153265.2042 | 189188.8271 | 826962.2559 | 737602.841 |
| 3-hydroxyheptanoic acid | 0 | 120448.5259 | 89903.78408 | 132304.0692 | 144087.7025 | 68638.86873 |
| 2-phenylacetamide | 9744.795471 | 2725524.85 | 2682551.536 | 1931010.806 | 2871040.281 | 2928970.721 |
| oleuropein | 0 | 280907.7286 | 243904.7982 | 268295.3894 | 218674.0312 | 246796.1799 |
| glu leu | 0 | 334963.9631 | 407161.4954 | 356970.8704 | 442152.9122 | 341751.8193 |
| vaccenic acid | 0 | 3976.72275 | 3120.18576 | 75320.52728 | 32208.88565 | 21601.85633 |
| salicyluric acid | 0 | 15531.30278 | 13517.85945 | 16944.35888 | 9765.481462 | 13946.55907 |
| met val | 0 | 992.4534386 | 0 | 315355.1141 | 0 | 368817.9222 |
| glutaminylhydroxyproline | 0 | 717542.2749 | 655450.6995 | 699596.5078 | 697896.3309 | 618069.987 |
| o-desmethylvenlafaxine glucuronide | 0 | 7451.468766 | 12012.47776 | 6417.653944 | 15458.68233 | 16182.28424 |
| trp ile | 447031.0468 | 6132.167278 | 9798.318709 | 220142.1458 | 11734.69381 | 207299.0668 |
| murocholic acid | 0 | 38209.20849 | 30863.41014 | 50376.73951 | 32470.1965 | 37012.27469 |
| γ-aminobutryic acid | 0 | 1507915.513 | 1485803.07 | 1405189.289 | 1929463.203 | 1321173.642 |
| l-β-aspartyl-l-phenylalanine | 0 | 526456.6389 | 460560.1218 | 655095.5049 | 398125.6372 | 626632.6349 |
| 2-pyridylacetic acid | 0 | 59336.26996 | 61696.33326 | 61294.51574 | 61294.32287 | 58657.65231 |
| valyl-phenylalanine | 0 | 9676.885997 | 1035.115024 | 685471.1745 | 1065.872807 | 613375.2436 |
| Uridine diphosphate-n-acetylglucosamine | 0 | 0 | 0 | 462342.0619 | 0 | 0 |
| noradrenochrome | 57866.82675 | 64631.79755 | 103252.0058 | 84534.89441 | 82593.39392 | 102932.3125 |
| glutamyl-hydroxyproline | 0 | 2134019.768 | 2069568.962 | 2168008.179 | 2035659.962 | 2119798.086 |
| 2-hydroxycinnamic acid | 0 | 20509152.54 | 20515394.35 | 14144232.36 | 22698076.89 | 20789517.36 |
| maleic acid | 16037.78848 | 13416.09663 | 12191.58077 | 13299.96586 | 12402.66044 | 10779.83608 |
| triphenylphosphine oxide | 0 | 317115.2079 | 335115.375 | 329249.6974 | 339862.5762 | 322402.963 |
| glycyl-isoleucine | 0 | 395330.572 | 106986.7805 | 5426687.647 | 181718.294 | 5799186.334 |
| gly thr leu | 0 | 342115.6601 | 407098.554 | 240646.8483 | 340296.6628 | 395129.4966 |
| ser trp | 219252.7064 | 33285.80896 | 28362.74075 | 209918.0008 | 28421.51447 | 309723.788 |
| cyclic 6-hydroxymelatonin | 769995.2988 | 325923.0611 | 325709.7286 | 276348.3976 | 292023.8973 | 293512.9123 |
| annosquamosin | 0 | 40680.15763 | 35259.43326 | 44904.43773 | 44029.57259 | 45593.05294 |
| val val | 6044308.036 | 148793.4297 | 38020.08233 | 7175793 | 69118.68973 | 6922507.944 |
| hydroxyprolyl-arginine | 0 | 8176469.425 | 8011209.367 | 8811244.17 | 8893394.027 | 9073590.32 |
| 4-guanidinobutanoic acid | 0 | 447352.3305 | 439789.6795 | 496435.8139 | 420758.35 | 433310.1359 |
| glutamylalanine | 0 | 864640.8203 | 879995.8234 | 7835654 | 932826.1879 | 967299.8847 |
| (γ-glutamyl-gamma-glutamyl)-s-methylcysteine | 0 | 222360.6964 | 253302.7593 | 264698.3665 | 193034.8317 | 179795.568 |
| ile glu leu | 0 | 608505.0075 | 956057.3425 | 292000.2529 | 356624.2551 | 866285.3841 |
| jurubine | 0 | 51233.94511 | 65058.70476 | 50363.20181 | 51828.01562 | 54631.48766 |
| val phe gly | 0 | 54597.61018 | 64286.44176 | 118407.4593 | 38882.92842 | 72924.35805 |
| plantagonine | 0 | 7106.186533 | 8425.542341 | 8345.854661 | 8643.073251 | 9001.528081 |
| melleolide | 0 | 4861.244392 | 24936.43928 | 7516.66622 | 9622.133504 | 11069.98002 |
| janthitrem | 0 | 418964.0901 | 339687.6363 | 355459.8608 | 356642.4421 | 371523.1241 |
| methylmalonic acid | 11093635.23 | 21756798.58 | 21056216.64 | 28607097.97 | 60522939.34 | 20690085.61 |
| 6-o-oleuropeoylsucrose | 0 | 249802.245 | 215670.6572 | 424813.8994 | 256264.2088 | 227561.9029 |
| n-(1-deoxy-1-fructosyl)valine | 0 | 834611.6431 | 922109.0066 | 988533.2498 | 1357945.981 | 1458491.977 |
| 7-methylguanosine | 0 | 103857.4303 | 112626.4529 | 6854.83317 | 0 | 55101.01685 |
| l-1,2,3,4-tetrahydro-β-carboline-3-carboxylic acid | 0 | 418423.3265 | 462991.1466 | 627095.851 | 267598.3706 | 355787.4288 |
| 4,4',5,7-tetrahydroxyflavan | 0 | 14998.29729 | 10662.71583 | 13853.58723 | 7043.04958 | 38259.34732 |
| 3,7-dihydroxy-2-phenyl-4h-chromen-4-one | 0 | 1928401.62 | 1861854.384 | 2106586.928 | 1587950.946 | 3214495.377 |
| curdione | 0 | 672.1147187 | 545.3340956 | 322.6511223 | 633.0899161 | 629.4773983 |
| leu asp phe | 658884.0073 | 227578.8377 | 261977.5819 | 218625.582 | 126060.6458 | 263410.2592 |
| ser gln ile | 0 | 212813.7101 | 278671.5994 | 151754.1355 | 243907.1968 | 251005.1782 |
| leu met | 2331009.705 | 26203.16312 | 0 | 2036816.034 | 3228.998772 | 1905647.32 |
| tyrosyl-alanine | 0 | 183263.9059 | 168784.41 | 203030.7336 | 169054.5949 | 189671.339 |
| guanosine | 2200060.595 | 4139090.677 | 4193723.149 | 606997.8862 | 0 | 15963.03743 |
| 2-keto-glutaramic acid | 0 | 109205.4759 | 114387.0108 | 79165.58422 | 139736.596 | 202919.8508 |
| (r)-(+)-2-pyrrolidone-5-carboxylic acid | 0 | 5559475.05 | 5753032.831 | 4386241.551 | 5873052.734 | 5989460.894 |
| jubanine | 0 | 70025.83005 | 68345.64679 | 316189.0781 | 351149.6381 | 5073.222064 |
| germacrenone | 0 | 25144.66029 | 20616.55009 | 22022.85529 | 21910.00189 | 21561.50225 |
| gly val | 0 | 23998.14634 | 37956.35378 | 91535.21359 | 51148.92032 | 32467.87388 |
| sucrose | 732339.5354 | 47529.0414 | 32458.5432 | 40867.31054 | 41172.74143 | 30990.60239 |
| 12-hydroxyheptadecanoic acid | 0 | 153764.6725 | 687246.444 | 380905.9958 | 313473.8031 | 152010.2324 |
| n-(1-deoxy-1-fructosyl)proline | 0 | 894429.0591 | 839143.4815 | 859179.7334 | 842354.8717 | 902443.5732 |
| n2,n2-dimethylguanosine | 0 | 165605.9266 | 231739.5445 | 13277.3552 | 457.6563385 | 383169.0781 |
| taurine | 58727.16132 | 54019.39536 | 57480.59168 | 56494.82291 | 38704.88828 | 46968.54708 |
| tryptophyl-proline | 0 | 413418.8622 | 414112.8 | 411809.6338 | 397689.7097 | 418383.6515 |
| gly leu | 0 | 500421.1212 | 305735.1703 | 9712002.416 | 402157.8113 | 12359618.94 |
| tyr tyr | 0 | 5605.499052 | 0 | 196636.0779 | 0 | 194079.6855 |
| tyr gly | 445916.996 | 47579.19741 | 9691.012525 | 341244.7028 | 14207.29749 | 612972.1855 |
| glutamylhistidine | 507532.1018 | 429356.8124 | 323397.0786 | 756814.9133 | 376393.8992 | 143432.1603 |
| 10-hydroxymelleolide | 0 | 43960.52513 | 48012.82409 | 50695.86846 | 49997.45519 | 48411.46985 |
| 2,3-dihydro-2,3-dihydroxy-4-(4-hydroxyphenyl)-1h-phenalen-1-one | 0 | 11515.2279 | 11935.1827 | 11482.14945 | 10467.79132 | 9890.947532 |
| 5-hydroxytryptophol | 0 | 128985.4916 | 125790.2158 | 94481.3641 | 131979.3497 | 142266.8252 |
| (s)-succinyldihydrolipoamide | 0 | 59464.79704 | 50494.01464 | 64563.17218 | 47238.65324 | 57282.79851 |
| nicotinamide | 1825000.313 | 83795.96443 | 79415.94824 | 4202931.652 | 126999.1218 | 148112.5775 |
| l-glutamic-γ-semialdehyde | 0 | 97312.09423 | 126709.9973 | 127687.3044 | 187398.7919 | 293063.2732 |
| traumatic acid | 42624.84076 | 62189.00777 | 48287.94626 | 47949.59769 | 44009.91963 | 64653.57319 |
| 18-oxocortisol | 0 | 1207976.714 | 1218171.044 | 967111.4353 | 1142188.074 | 1060925.09 |
| bis(2-ethylhexyl) phthalate | 0 | 577190.8838 | 400049.0349 | 480404.2702 | 447462.5047 | 353403.0117 |
| capsidiol | 25992.09274 | 1796.904636 | 2304.031752 | 2889.605785 | 2332.964194 | 2072.827243 |
| pseudoecgonine | 0 | 133976.0906 | 119801.2174 | 147757.7223 | 156527.8537 | 142630.7722 |
| tyr leu | 559228.8165 | 14961.20398 | 16842.69028 | 368823.8702 | 27366.37906 | 402023.1372 |
| thr met | 0 | 0 | 0 | 218173.9097 | 0 | 297335.5995 |
| 5-[(6-hydroxy-3,7-dimethylocta-2,7-dien-1-yl)oxy]-7-methoxy-2h-chromen-2-one | 0 | 679372.3244 | 620944.0272 | 677598.5349 | 593533.7575 | 747626.6755 |
| ala gly ile | 1421278.202 | 488857.866 | 654154.9522 | 138458.6101 | 557732.2512 | 614948.5501 |
| phe thr gly | 0 | 94503.96377 | 124801.3554 | 138267.063 | 109334.3122 | 126390.7999 |
| ile leu gly | 82386.75479 | 70873.47842 | 111649.6711 | 171265.0487 | 78604.02378 | 124885.025 |
| capryloylglycine | 0 | 29456.99505 | 36000.23736 | 32622.29201 | 33485.85867 | 29029.10614 |
| hydroxyisonobilin | 0 | 44330.64633 | 43327.67398 | 39937.14358 | 44764.61691 | 34952.51575 |
| flazine | 428926.132 | 199665.828 | 148459.0455 | 190754.735 | 287861.3455 | 286170.8643 |
| phe gly | 2831220.988 | 156164.396 | 88039.8501 | 1763649.11 | 107638.3162 | 2486984.867 |
| valyl-valine | 0 | 21961.90312 | 0 | 1921928.202 | 7537.041214 | 1902783.888 |
| valyl-tryptophan | 0 | 10109.66162 | 5602.264784 | 587169.8669 | 9829.307298 | 488201.0557 |
| d-arginine | 0 | 1171759.988 | 1115862.571 | 1073641.133 | 38501.59969 | 1062221.159 |
| ophiopogonin c' | 0 | 4863729.889 | 4703116.805 | 5215871.63 | 4999279.968 | 4953588.938 |
| val asn glu | 0 | 641753.7738 | 664586.9613 | 782598.3082 | 476648.9319 | 901664.0563 |
| ala-val-oh | 0 | 257553.2361 | 251671.0304 | 589541.5145 | 295336.0876 | 506091.3631 |
| deoxypyridinoline | 3404319.393 | 442733.728 | 452172.4394 | 457909.3159 | 513603.3776 | 488971.7685 |
| 5,10-pentadecadien-1-ol | 0 | 39300.5765 | 37213.85873 | 45340.20129 | 41106.14866 | 40353.93956 |
| 2',3',4'-trihydroxyacetophenone | 22915.55451 | 63784.15208 | 51849.32449 | 34517.36063 | 75329.96398 | 75579.57881 |
| bβ-alanyl-l-lysine | 905194.448 | 611121.0537 | 597629.8253 | 624708.3996 | 188616.3852 | 1066339.195 |
| val ile ala | 0 | 82533.82308 | 140250.029 | 133962.2394 | 73751.67933 | 129681.4203 |
| isepamicin | 1106270.869 | 566528.6329 | 576987.929 | 474478.7163 | 611031.4729 | 313673.4357 |
| arg leu | 0 | 391829.0684 | 372646.9081 | 2642630.168 | 198217.0893 | 7894608.297 |
| n-(1-deoxy-1-fructosyl)tyrosine | 0 | 3823735.036 | 3767025.224 | 3751203.942 | 4497063.626 | 4010590.806 |
| pro gly val | 0 | 471697.4178 | 470283.6087 | 569070.7169 | 518745.1113 | 517985.1547 |
| 2-acetamido-4-methylphenyl acetate | 0 | 921120.0257 | 954475.1891 | 926320.7214 | 956491.4177 | 862072.8397 |
| 1-cyclopropyl-4-methyl-1,3-cyclohexanediol | 0 | 83522.48358 | 90684.28695 | 72123.25893 | 68833.2162 | 68425.28481 |
| ala val thr | 0 | 1174453.036 | 1484581.432 | 1741067.99 | 1487631.305 | 1526778.348 |
| 2-undecen-1-ol | 0 | 61074.8731 | 52173.57469 | 34867.05149 | 78639.74633 | 75504.5847 |
| l-n-(3-carboxypropyl)glutamine | 0 | 1404875.691 | 1243072.193 | 1349767.461 | 1352313.198 | 1368063.466 |
| 10,20-dihydroxyeicosanoic acid | 0 | 147055.0466 | 68120.27605 | 165948.0769 | 122714.3935 | 158574.7401 |
| leu-leu-leu | 0 | 163912.6228 | 158111.7812 | 176353.4448 | 159052.0354 | 172864.8286 |
| ethotoin | 0 | 71399.02355 | 64139.67936 | 63394.46666 | 57630.16585 | 53040.15257 |
| β-casomorphin | 0 | 1946465.311 | 1920480.163 | 1760594.978 | 2277343.78 | 1751388.43 |
| gly pro ala | 328514.6028 | 117274.0421 | 126272.9397 | 138978.1214 | 129385.9123 | 128683.922 |
| 6-hydroxy-1h-indole-3-acetamide | 0 | 17481.95599 | 34829.48806 | 40858.14003 | 22889.33542 | 30458.15149 |
| 4-methyl-5-thiazoleethanol | 0 | 145257.2034 | 158443.0152 | 168862.7287 | 76243.34469 | 202891.1124 |
| α-methyltryptamine | 0 | 87994.5327 | 91585.62962 | 91725.14387 | 108783.1796 | 133246.7814 |
| benzaldehyde | 0 | 841223.6707 | 813532.3784 | 1009187.866 | 827335.3945 | 833440.6284 |
| n-formyl-4-amino-5-aminomethyl-2-methylpyrimidine | 0 | 195798.0017 | 195760.814 | 181715.642 | 184519.1876 | 174565.7225 |
| ala gly val | 0 | 1373330.971 | 1682075.3 | 676702.1019 | 1489604.921 | 1585195.939 |
| o-tyrosine | 0 | 2005076.335 | 1492973.724 | 2414208.386 | 1967290.149 | 1510198.426 |
| cotinine glucuronide | 0 | 85456.29824 | 67601.77657 | 64522.65107 | 100063.3202 | 100049.7552 |
| phenylalanyl-valine | 0 | 25509.70679 | 15018.62857 | 662447.9321 | 27518.28793 | 527914.2047 |
| 10-hydroxy-8-nor-2-fenchanone glucoside | 0 | 270670.3701 | 54531.29751 | 168308.5296 | 295064.4896 | 99777.8836 |
| gly gly pro val | 0 | 5488865.519 | 6819136.891 | 8173647.949 | 6759160.689 | 7829478.63 |
| pectachol | 0 | 331817.3845 | 212525.5205 | 426770.0922 | 418500.5555 | 374273.5041 |
| α-aminodiphenylacetic acid | 168623.9606 | 58469.2644 | 49107.96333 | 49017.89895 | 78682.8748 | 75033.0505 |
| phenylalanyl-lysine | 0 | 0 | 0 | 1653353.973 | 0 | 2779198.754 |
| 21-deoxycortisol | 0 | 739337.1536 | 802214.5803 | 719701.1339 | 818810.0062 | 761091.5116 |
| glucosamine | 0 | 354289.5844 | 362885.0446 | 405528.8082 | 470102.011 | 551563.6871 |
| 6'-hydroxy-o-desmethylangolensin | 0 | 56698.98131 | 37484.57446 | 45778.56222 | 31295.1434 | 94635.28913 |
| cytosine | 375559.8066 | 1485047.199 | 1483511.388 | 1248738.735 | 345851.1823 | 285832.8534 |
| 2-methylguanosine | 0 | 51571.92224 | 58966.86229 | 0 | 0 | 24947.87977 |
| 2'-aminoacetophenone | 0 | 173132.8508 | 201017.6305 | 140889.9399 | 347136.7045 | 284531.023 |
| ile gly thr gly | 1274249.498 | 143146.4959 | 171822.3687 | 194684.0336 | 165498.8451 | 162859.8316 |
| pa(18:3(9z,12z,15z)/20:5(5z,8z,11z,14z,17z)) | 0 | 75433.56959 | 88427.5163 | 75857.31428 | 83203.04356 | 81499.90051 |
| asparaginyl-isoleucine | 0 | 932394.6705 | 767427.8257 | 1209481.595 | 1023473.855 | 961498.9071 |
| 12'-apo-b-carotene-3,12'-diol | 0 | 47946.032 | 43263.36611 | 56730.24138 | 47849.25304 | 43822.8991 |
| angiotensin iv | 0 | 451086.9142 | 373442.0975 | 623048.1754 | 410758.0945 | 446737.3311 |
| p-tolyl sulfate | 528528.4877 | 328188.1246 | 342708.5028 | 402660.9792 | 336433.4222 | 349027.8296 |
| glutaminylglycine | 129715.6575 | 35656.79701 | 41214.12839 | 18224.30212 | 61960.94639 | 81223.73403 |
| acetylhomoserine | 0 | 54651.77605 | 57749.8231 | 61772.17507 | 53946.94481 | 59854.59947 |
| 20-trifluoro-ltb4 | 138118.7682 | 277812.2528 | 256917.6984 | 248350.6378 | 267599.7058 | 249895.8321 |
| ethyl cellulose | 0 | 455594.618 | 497418.0511 | 561196.6024 | 484193.5555 | 602395.8541 |
| ser ile gly | 0 | 172531.2786 | 254682.0263 | 336992.3307 | 255673.9514 | 251988.4008 |
| asp val | 0 | 492070.2482 | 473923.8803 | 765595.2424 | 479165.6006 | 712759.9243 |
| asp ile ser | 846135.8922 | 416747.5232 | 432473.1508 | 495111.6444 | 375922.9485 | 429862.4826 |
| perlolyrine | 0 | 8884.490713 | 5940.844606 | 8465.038695 | 15583.276 | 16924.62308 |
| n-acetyl-b-glucosaminylamine | 0 | 443123.8007 | 466756.1918 | 361154.3018 | 478641.1711 | 500165.1471 |
| gly tyr | 0 | 1115122.97 | 573918.0538 | 1183281.697 | 516650.7179 | 1707459.571 |
| gln val asp glu | 227019.9638 | 146154.601 | 148789.7026 | 112042.0666 | 111495.4808 | 117120.3804 |
| n6,n6-dimethyladenosine | 0 | 64965.26754 | 90933.44882 | 351.2875927 | 1465.384053 | 41247.39374 |
| thr lys thr thr | 1766479.863 | 551002.4123 | 556533.6837 | 619902.6676 | 691896.0927 | 639195.2785 |
| cl(8:0/10:0/18:2(9z,11z)/20:0) | 0 | 3824110.679 | 2957534.877 | 1793041.228 | 2049965.438 | 2704859.301 |
| l-menthyl acetoacetate | 0 | 21637.16556 | 26761.61425 | 23424.63502 | 22189.64179 | 19813.28401 |
| γ-glutamylalanine | 0 | 211737.0015 | 396986.98 | 130927.3361 | 240961.551 | 130425.404 |
| capsianoside | 0 | 130606.3478 | 189187.199 | 166683.9044 | 211182.7205 | 104891.741 |
| glucoconvallasaponin | 0 | 1081256.56 | 974618.4698 | 1335656.192 | 1151754.647 | 1055729.655 |
| γ-glutamyl-s-methylcysteinyl-beta-alanine | 0 | 111460.8425 | 107068.7705 | 146417.2055 | 99596.72811 | 129033.3221 |
| 3-furoic acid | 644551.8856 | 400510.4871 | 389846.6424 | 453683.9594 | 2563.850129 | 19754.94868 |
| (2e,11z)-wyerone acid | 0 | 1106061.409 | 960464.8135 | 1012508.938 | 730027.5075 | 1443524.455 |
| thr val thr | 665928.9045 | 88114.58185 | 91303.66783 | 103449.2005 | 88168.44829 | 91503.45447 |
| asn leu | 0 | 526766.0978 | 555089.0535 | 1969932.988 | 570642.5028 | 3646218.777 |
| 12-hydroxy-10e-octadecenoic acid | 0 | 779.3342885 | 269.8888429 | 1700.325105 | 16245.62194 | 2071.583681 |
| hydroxyprolyl-lysine | 0 | 36357.75376 | 50032.82738 | 15201.90315 | 46340.02771 | 45990.15707 |
| 5-nitro-2-phenylpropylaminobenzoic acid | 0 | 34414.59653 | 29027.59351 | 31897.23143 | 31440.59107 | 35204.23551 |
| phenylalanyl-isoleucine | 0 | 26855.61944 | 24494.18682 | 20633.95637 | 27531.21055 | 27226.89493 |
| val ala val | 0 | 122701.5081 | 193896.1303 | 52694.89234 | 83748.54651 | 192874.717 |
| lys leu | 0 | 50731.124 | 81717.61244 | 5729.860171 | 3938.893732 | 75021.10548 |
| tyrosyl-phenylalanine | 0 | 115817.4393 | 120632.7212 | 121035.1729 | 110633.7815 | 115456.8634 |
| tyr gln | 0 | 26172.80426 | 24502.48656 | 287094.2802 | 52025.04586 | 422732.7901 |
| isoleucyl-arginine | 3649673.355 | 1434963.442 | 1524406.386 | 1588446.026 | 1538499.338 | 1537330.021 |
| ser his | 0 | 90348.20195 | 52484.89207 | 160985.6381 | 21695.18051 | 243609.8754 |
| (1xi,3xi)-1,2,3,4-tetrahydro-1-methyl-beta-carboline-3-carboxylic acid | 0 | 355043.7371 | 186548.6702 | 1837888.088 | 314861.3645 | 4340948.846 |
| glycyl-methionine | 0 | 6714.849975 | 0 | 164120.6936 | 8542.927402 | 389933.9025 |
| pa(18:3(9z,12z,15z)/20:4(8z,11z,14z,17z)) | 0 | 122166.8528 | 244470.2217 | 180123.5337 | 209222.6777 | 212466.6065 |
| 3-ethenylphenol | 0 | 58677328.25 | 46705060.44 | 21080654.93 | 46766086.47 | 44204782.79 |
| 4-(2,6,6-trimethyl-1,3-cyclohexadien-1-yl)-2-butanone | 0 | 19252.0624 | 6350.315524 | 3035.582306 | 5421.22373 | 7627.017841 |
| isovalerylglutamic acid | 0 | 60463.97726 | 59314.12347 | 69958.64691 | 64554.13778 | 79929.95607 |
| 2-descarboxy-betanidin | 285834.8467 | 31493.00656 | 30907.43209 | 53229.57758 | 38686.14908 | 38553.63967 |
| nerol | 0 | 125342.904 | 240771.1819 | 538798.3245 | 87540.17292 | 606664.9092 |
| n-acetylisoleucine | 0 | 579419.5537 | 963109.8418 | 182349.9327 | 674655.6747 | 269437.4701 |
| mg(14:1(9z)/0:0/0:0) | 0 | 26088.08523 | 23621.89491 | 26400.10142 | 20929.07163 | 28817.85873 |
| aspartylphenylalanine | 0 | 161213.5006 | 135447.16 | 153435.5991 | 143064.6037 | 171080.3187 |
| val phe | 1727356.595 | 27759.89797 | 9086.827172 | 1359220.974 | 9930.193019 | 1242135.053 |
| glycochenodeoxycholic acid 3-glucuronide | 0 | 514769.7635 | 533208.081 | 477724.1022 | 533651.4064 | 447364.3555 |
| n-carbamylglutamate | 0 | 4629.653894 | 6953.649163 | 4035.444059 | 90211.25412 | 91965.47235 |
| val glu gly | 79068.26291 | 161285.8418 | 143306.8948 | 288145.8004 | 145450.2006 | 182311.309 |
| 2-dodecylbenzenesulfonic acid | 0 | 3058309.351 | 2927476.08 | 3274075.301 | 3019892.579 | 2784593.598 |
| prednisone | 0 | 583086.6954 | 596154.4262 | 528324.9857 | 495702.9956 | 889206.9184 |
| ps(20:5(5z,8z,11z,14z,17z)/14:1(9z)) | 0 | 26094.38595 | 30269.54832 | 28619.01098 | 24361.42497 | 28039.27288 |
| guanosine monophosphate | 0 | 3907321.865 | 3395148.993 | 3747279.467 | 3505753.992 | 3362098.086 |
| phenylalanyl-histidine | 0 | 2313514.266 | 2537863.848 | 2693167.114 | 2557173.979 | 2347987.243 |
| ala leu ala | 0 | 104233.8237 | 191613.0752 | 133183.4785 | 109476.1652 | 187038.3546 |
| n-(1-deoxy-1-fructosyl)histidine | 0 | 131957.4402 | 80935.43196 | 104784.782 | 75522.4521 | 76214.75659 |
| citroside | 0 | 227103.0564 | 261540.4857 | 400179.8251 | 293866.2009 | 285233.0432 |
| pimecrolimus | 0 | 101165.8508 | 288033.155 | 406531.0724 | 595815.525 | 13139.14211 |
| pro gln | 0 | 583896.8123 | 790057.2888 | 655351.2493 | 853042.0804 | 401226.6072 |
| acetyl-dl-valine | 0 | 10105.47562 | 22302.05896 | 9170.848098 | 15774.91327 | 7643.639633 |
| pyroglutamylvaline | 0 | 925649.4041 | 681739.8468 | 16068109.54 | 766380.0741 | 954417.1527 |
| aspartyl-tyrosine | 0 | 95469.10633 | 89033.75115 | 98192.72056 | 99344.81321 | 111747.643 |
| tyrosyl-γ-glutamate | 0 | 0 | 0 | 0 | 0 | 49577.84716 |
| ophthalmic acid | 0 | 1399278.102 | 1262159.154 | 1395828.179 | 1157149.842 | 1524956.634 |
| 5-methoxytryptophan | 0 | 435733.5386 | 581745.4949 | 634002.3012 | 308428.1136 | 347683.7438 |
| val leu ser | 0 | 153164.9868 | 243306.9913 | 264880.7859 | 139787.3287 | 213490.517 |
| telbivudine | 0 | 816812.3592 | 854979.0773 | 683498.0748 | 1019433.771 | 983692.9678 |
| pro leu pro tyr | 0 | 443636.8289 | 461754.6841 | 370094.1587 | 419084.9921 | 829682.8694 |
| serylisoleucine | 0 | 51411.93815 | 15151.45135 | 1984505.231 | 24113.91965 | 2860915.82 |
| ile thr ile | 739150.5818 | 251517.748 | 390818.4707 | 149502.5211 | 212005.3026 | 350019.3837 |
| vidarabine | 0 | 49515454.42 | 49893026.38 | 3837891.462 | 45448.15451 | 129544.2786 |
| threoninyl-γ-glutamate | 920883.7198 | 10478835.47 | 10576420.39 | 9160878.999 | 11856203.66 | 12193598.4 |
| cis-zeatin-7-n-glucoside | 0 | 47734.68676 | 62347.40159 | 48094.91079 | 45408.94606 | 183362.973 |
| 1-(1,2,3,4,5-pentahydroxypent-1-yl)-1,2,3,4-tetrahydro-beta-carboline-3-carboxylate | 0 | 3703741.953 | 3981488.9 | 5164890.471 | 5201058.971 | 4535875.92 |
| 3-oxoglutaric acid | 0 | 215765582.3 | 207793799.1 | 221550191.6 | 1106309.493 | 15970342.09 |
| asp ser leu | 179014.4813 | 156445.3369 | 147116.0215 | 157610.5402 | 144751.7162 | 189502.6658 |
| dihydrocoumarin | 1393921.432 | 2068472.026 | 1980587.223 | 2397879.462 | 2086999.915 | 2011651.673 |
| 5-methyl-2-furaldehyde | 406641.7034 | 475308.856 | 522559.7958 | 342696.443 | 532999.686 | 502994.3801 |
| α-(methylenecyclopropyl)glycine | 0 | 1428020.199 | 262566.1096 | 281130.3832 | 283013.3615 | 1914874.23 |
| menthone 1,3-glyceryl ketal | 0 | 0 | 0 | 0 | 39295.33011 | 5884.104623 |
| (r)-mandelic acid | 34255.28928 | 23406.74943 | 27244.21789 | 26491.80352 | 21936.88458 | 28843.20877 |
| 1-(β-d-ribofuranosyl)-1,4-dihydronicotinamide | 0 | 386175.1555 | 434885.2558 | 784738.0759 | 310193.9772 | 1084204.895 |
| val ile thr | 1289586.071 | 328492.072 | 488546.7371 | 499195.9588 | 221731.9399 | 532668.7104 |
| 5-methoxytryptophol | 0 | 533407.2355 | 408421.0855 | 511176.2068 | 469181.3697 | 397890.0266 |
| canavalioside | 0 | 311348.7103 | 324139.0064 | 252176.4321 | 388215.2937 | 358139.7479 |
| 5-phenyl-1,3-oxazinane-2,4-dione | 25638.95813 | 87716.7582 | 46149.64395 | 54952.46771 | 65129.65594 | 58156.21319 |
| leucyl-leucine | 0 | 11128.81826 | 8067.711892 | 821274.9569 | 2564.801262 | 656875.2252 |
| n-(4-aminobutyl)-3-(4-hydroxyphenyl)prop-2-enimidic acid | 0 | 22553.11504 | 15479.36147 | 680563.8603 | 0 | 543064.9091 |
| benzeneacetonitrile | 0 | 821802.8194 | 815921.3188 | 996141.3511 | 817169.8497 | 819691.6563 |
| ala ser ile | 747004.1111 | 311217.1437 | 425296.2779 | 238961.5677 | 390103.2142 | 410404.7945 |
| 3-hydroxytridecanoic acid | 0 | 0 | 0 | 0 | 23926.30146 | 0 |
| paromomycin | 0 | 1823119.723 | 1541651.857 | 1878907.476 | 1921195.452 | 1493371.524 |
| nicotyrine | 0 | 1250652.289 | 1393937.617 | 1528940.564 | 1478768.223 | 965830.9038 |
| asp asp ile | 0 | 71245.42882 | 79698.6007 | 99227.52045 | 81683.14226 | 174219.7904 |
| 3,5,7-trihydroxy-2-(3-hydroxy-5-methoxyphenyl)-4h-chromen-4-one | 0 | 21959.66941 | 19795.34975 | 21333.95883 | 18194.49844 | 22885.66463 |
| ile ala ile | 0 | 418022.344 | 762265.5358 | 213601.5462 | 297264.3655 | 700439.7337 |
| 1-acetylindole | 0 | 17877.15845 | 6410.610002 | 4498.42998 | 7799.468343 | 6355.482065 |
| val ile | 0 | 91829.25327 | 21292.08442 | 3214054.227 | 23332.17594 | 4708572.582 |
| leucyl-hydroxyproline | 0 | 144010.3567 | 190349.7068 | 114193.5732 | 104520.9982 | 182518.6194 |
| pro pro phe | 458432.9471 | 354375.6092 | 418758.483 | 431623.6262 | 428589.2973 | 435944.7596 |
| asp ser ser thr | 0 | 40999.06511 | 32813.86683 | 0 | 7508.63786 | 130099.3414 |
| γ-glutamyltryptophan | 475195.8555 | 11015.33352 | 0 | 78834.89616 | 2810.782482 | 2811.65255 |
| hexadecanedioic acid | 0 | 12539.56194 | 19985.71064 | 1652.828436 | 37658.44081 | 115499.9771 |
| deterrol stearate | 0 | 48078.86845 | 38197.51788 | 60926.86594 | 52086.66022 | 50729.86807 |
| l,l-cyclo(leucylprolyl) | 544467.5101 | 448209.0412 | 464746.2933 | 486487.0097 | 445728.6213 | 537228.3283 |
| histidinyl-tyrosine | 0 | 164748.8682 | 165594.5937 | 38322.32276 | 81810.57158 | 1146475.669 |
| icariside | 0 | 172447.8788 | 168904.3885 | 138777.083 | 165948.3295 | 170187.1423 |
| asp leu ser glu | 891955.6577 | 616465.4883 | 578557.4328 | 649653.0815 | 610439.6967 | 519978.3397 |
| histidinyl-glycine | 0 | 391229.3975 | 421261.6014 | 424170.8028 | 453856.2115 | 374421.1185 |
| ginsenoyne | 0 | 136751.0869 | 125243.6105 | 134584.0898 | 124134.7185 | 130697.7823 |
| niazimin | 0 | 205598.1855 | 175420.0743 | 188672.2269 | 178408.6512 | 202974.9738 |
| (2s,3's)-alpha-amino-2-carboxy-5-oxo-1-pyrrolidinebutanoic acid | 0 | 247763.6146 | 179467.8664 | 3022915.747 | 187238.2495 | 171630.372 |
| gly leu phe | 66594.66992 | 245699.1261 | 430131.0401 | 381837.1338 | 430113.6731 | 394475.7074 |
| asn glu leu | 0 | 286692.3905 | 322399.5202 | 201418.5447 | 314225.21 | 346033.6236 |
| apgpr enterostatin | 0 | 1407000.762 | 1380797.082 | 530997.323 | 1889314.834 | 1451807.593 |
| 5a,6a-epoxy-7e-megastigmene-3b,9e-diol 9-glucoside | 0 | 285854.6611 | 314147.5655 | 253193.8787 | 295726.0058 | 282988.3079 |
| 1-(m-methoxycinnamoyl)pyrrolidine | 0 | 182790.1872 | 139741.6483 | 147303.787 | 142555.5726 | 189806.8249 |
| 8-azaspiro[4.5]decane-8-butanoic acid, 7,9-dioxo(buspirone metabolite) | 0 | 16585.52111 | 15561.93348 | 22793.4281 | 14381.15815 | 15647.93248 |
| 1-(5-acetyl-2-hydroxyphenyl)-3-methyl-1-butanone | 0 | 26462.46223 | 37841.79372 | 37161.5714 | 36770.0916 | 38140.06467 |
| withaperuvin | 0 | 118635.8226 | 99966.56296 | 126025.1856 | 123642.4801 | 138100.4127 |
| gly leu ala | 2913599.43 | 1595115.043 | 2276106.101 | 2226139.647 | 2207216.563 | 2147923.76 |
| cytidine | 0 | 13492.86245 | 13545.80078 | 7916.452393 | 15393.186 | 11315.09343 |
| m-xylene | 0 | 5312.696791 | 3151.153338 | 6478.897166 | 6414.577335 | 6239.915438 |
| n-(3-indolylacetyl)-l-isoleucine | 0 | 18499.9002 | 16940.49935 | 0 | 12568.36828 | 20715.45692 |
| phenylalanyl-aspartate | 0 | 24911.30064 | 9929.138565 | 585724.9504 | 9403.685821 | 927132.4808 |
| cl(8:0/11:0/18:2(9z,11z)/19:0) | 0 | 1189246.943 | 989930.4627 | 797233.612 | 1022866.769 | 835908.2755 |
| saccharopine | 0 | 192088.3383 | 177626.5401 | 155656.2541 | 202975.603 | 175682.2113 |
| valyl-arginine | 1171872.374 | 2214743.557 | 2212438.237 | 2172395.981 | 2213441.637 | 2252758.558 |
| tsibulin | 0 | 7748.468222 | 4953.12942 | 25529.5695 | 42764.34041 | 31165.43776 |
| dihydrozeatin-o-glucoside | 0 | 141464.0147 | 174749.7383 | 130385.9175 | 139339.9844 | 134600.3086 |
| cyclo(deltaala-l-val) | 0 | 5221651.414 | 5104771.948 | 5516510.163 | 4901140.985 | 5782541.082 |
| 3'-amp | 0 | 4501407.765 | 5185456.994 | 14430660.07 | 13751024.82 | 8267167.172 |
| anthranilic acid | 11468.88678 | 8367.575978 | 9988.075521 | 5193.229898 | 3999.536311 | 7944.974967 |
| pro pro tyr ser | 65082.52838 | 51775.36211 | 53745.39175 | 50175.95643 | 49620.74111 | 50243.93358 |
| d-xylono-1,5-lactone | 0 | 3358143.868 | 2149550.083 | 937187.5455 | 3952431.112 | 2340899.957 |
| 1-deoxy-1-(n6-lysino)-d-fructose | 0 | 1676423.836 | 1408682.98 | 1497735.058 | 1382766.576 | 1826382.21 |
| 5-methylthioribose | 0 | 34721.51212 | 28508.55635 | 19965.24957 | 26041.77324 | 30672.38225 |
| leucyl-histidine | 0 | 865732.4727 | 862111.7973 | 885766.6938 | 914550.6495 | 866880.1854 |
| toluene | 0 | 4270.327387 | 1452.449706 | 87274.26202 | 5127.226282 | 8518.714729 |
| ile ile | 2251380.708 | 30915.96415 | 14102.43398 | 1695307.635 | 8308.912719 | 1282583.062 |
| phosphocholine | 0 | 44225.15233 | 39096.55885 | 56135.4453 | 57145.79097 | 46924.06186 |
| threoninyl-lysine | 0 | 293114.7885 | 235583.4907 | 296345.1686 | 140519.1308 | 426821.9092 |
| l-carnitine | 0 | 576579.6037 | 600866.0333 | 659242.0283 | 594775.9043 | 525954.5955 |
| leucyl-valine | 0 | 74849.72892 | 26922.95957 | 1979264.915 | 28678.60047 | 2372564.457 |
| 2,5-dimethylpyrazine | 0 | 154661.55 | 166807.6861 | 109631.1966 | 161933.2416 | 136680.4984 |
| asp ile thr asp | 0 | 176121.8804 | 165591.2681 | 201019.0042 | 189298.1409 | 186123.1091 |
| glycocholic acid | 64160.88397 | 315851.3067 | 304131.5231 | 52138.1381 | 323961.4609 | 271213.0052 |
| l-trans-5-hydroxy-2-piperidinecarboxylic acid | 0 | 2542283.577 | 2552641.923 | 2216817.592 | 2824693.529 | 2503023.207 |
| 6-hydroxyindolelactate | 0 | 39562.67719 | 9309.661394 | 8580.13889 | 12723.35943 | 5582.816655 |
| ala ile | 0 | 71131.68275 | 12263.82913 | 2904017.469 | 19321.68517 | 4281101.256 |
| lysope(18:4(6z,9z,12z,15z)/0:0) | 0 | 308087.769 | 459775.8943 | 268435.149 | 358654.3634 | 134790.6131 |
| 2-ethyl-2-hydroxybutyric acid | 0 | 117872752 | 92271066.55 | 15679067.96 | 87621113.49 | 73805438.91 |
| glutethimide | 0 | 62464.37982 | 35605.89391 | 49430.26779 | 42097.19186 | 67938.97404 |
| dihydrofolic acid | 0 | 67150.2485 | 46856.6093 | 63862.15305 | 52357.65293 | 73397.29616 |
| n-lactoyl-tyrosine | 0 | 261668.2035 | 1052110.466 | 127959.6555 | 258045.9365 | 151230.9299 |
| tyr met | 0 | 0 | 458.5243612 | 78088.69922 | 6.909382917 | 150970.0525 |
| pro gly pro phe | 0 | 412115.1085 | 402728.838 | 402670.476 | 394277.2247 | 586232.2351 |
| amlexanox | 0 | 8225.934986 | 18156.78818 | 26179.712 | 6936.477776 | 5019.037815 |
| histidinyl-proline | 16530133.33 | 11307093.12 | 11506665.59 | 11767528.74 | 13889235.73 | 13238917.12 |
| isopropyl β-d-glucoside | 0 | 6939.285896 | 5066.011024 | 5574.788628 | 8391.154762 | 7181.090004 |
| threoninyl-valine | 0 | 665521.5542 | 435715.0155 | 6422498.976 | 408523.9945 | 7662676.78 |
| 2,3-dihydro-5-(3-hydroxypropanoyl)-1h-pyrrolizine | 80141.42421 | 14547.48669 | 16935.1443 | 9257.662075 | 17911.70966 | 18008.29491 |
| tyrosyl-isoleucine | 0 | 3007.093739 | 4185.310823 | 199353.1251 | 4280.840022 | 182514.1159 |
| glutaminylphenylalanine | 0 | 127478.5584 | 144341.7078 | 177430.0886 | 114757.4266 | 191089.7106 |
| sterol | 0 | 83609.20122 | 107048.8426 | 89851.90363 | 49174.05124 | 85999.04822 |
| val asp val | 0 | 330855.6782 | 350306.4086 | 270215.0145 | 229175.4954 | 400622.0427 |
| l-agaritine | 0 | 565738.5701 | 531682.5173 | 584940.0974 | 480750.1639 | 578153.1122 |
| leu phe | 0 | 16181.21326 | 8591.277964 | 1112645.137 | 16095.29047 | 778228.1528 |
| deoxycytidine | 0 | 85747.46726 | 78286.41967 | 205241.2409 | 158653.1289 | 105724.5306 |
| ethyl aconitate | 0 | 7159.358395 | 62804.90508 | 7720.661401 | 36983.72053 | 0 |
| isopropyl apiosylglucoside | 0 | 18975.71588 | 16959.11326 | 13977.33442 | 15702.68627 | 15944.54188 |
| 5-nonyltetrahydro-2-oxo-3-furancarboxylic acid | 0 | 89272.11101 | 79092.01185 | 82318.44422 | 83081.55021 | 80404.54297 |
| gypsogenin 3-o-b-d-glucuronide | 0 | 336801.1019 | 392041.568 | 308522.2308 | 326615.8695 | 312440.3055 |
| gly his | 0 | 354720.5289 | 300871.4157 | 305661.6894 | 255003.0081 | 374773.6802 |
| n-methylanthranilic acid | 0 | 60989.86167 | 55106.36526 | 60039.6447 | 58958.98512 | 55668.11608 |
| cucurbitacin | 0 | 200553.9781 | 197193.8877 | 209079.536 | 224095.8615 | 215022.0242 |
| ile val ser | 0 | 279583.3156 | 419543.109 | 492769.5181 | 237113.8947 | 388331.5953 |
| netilmicin | 0 | 3120835.373 | 2992283.692 | 3538195.02 | 3237264.489 | 3274370.128 |
| 4-acetyl-2-prenylphenol glucoside | 0 | 192843.481 | 252607.6502 | 223230.9626 | 264578.4737 | 293911.9132 |
| n-lactoyl-leucine | 0 | 306292.6373 | 260081.918 | 251173.2751 | 275822.2994 | 278647.6812 |
| β-alanyl-l-arginine | 0 | 113965.4963 | 129777.0246 | 98468.91058 | 0 | 241593.1412 |
| citrulline | 0 | 1330180.096 | 1237393.199 | 1122486.444 | 1100920.186 | 1123874.609 |
| 2'-o-methyladenosine | 0 | 8148.431488 | 9796.186008 | 40286.95739 | 15151.91566 | 62064.968 |
| phenylalanyl-methionine | 0 | 46712.03632 | 32271.9298 | 49477.723 | 41202.58771 | 56816.61447 |
| lys leu glu | 0 | 361000.8827 | 683125.7423 | 175673.2016 | 79871.08632 | 624588.8505 |
| gly asp phe | 0 | 237397.218 | 276519.0248 | 219711.4203 | 273332.6691 | 277236.1926 |
| ps(15:0/18:2(9z,12z)) | 0 | 67707.33413 | 55476.64462 | 146082.1825 | 42719.10493 | 53937.1589 |
| udp-d-galactose | 0 | 0 | 0 | 251444.2697 | 0 | 0 |
| phe pro ile | 0 | 473650.3449 | 647931.7451 | 304818.4646 | 377078.6667 | 563484.9321 |
| leu pro ile | 0 | 853765.1502 | 1125974.53 | 344415.8225 | 546318.1539 | 1109223.513 |
| lipoyllysine | 0 | 26465.03851 | 26680.33169 | 36968.64938 | 0 | 50744.75351 |
| 5-hydroxyprimaquine | 0 | 67896.50414 | 62088.20499 | 58896.68229 | 59708.33804 | 59274.22239 |
| 5-methoxysalicylic acid | 0 | 5887.352347 | 7458.371027 | 5712.283514 | 5973.236675 | 5667.520299 |
| sclareol | 0 | 1764.792267 | 0 | 12092.56164 | 39340.92766 | 94923.14017 |
| Dihydroxy-dimethoxycyclohexylidene-acetonitrile | 0 | 272899.7335 | 267396.3735 | 276010.4618 | 255567.2668 | 283683.0517 |
| 2-aminoheptanedioic acid | 0 | 1940384.819 | 1711055.36 | 1669003.604 | 2247485.286 | 1775973.319 |
