# Supplementary material for: Lacticaseibacillus rhamnosus C1 effectively inhibits Penicillium roqueforti: Effects of antimycotic culture supernatant on toxin synthesis and corresponding gene expression
Source: Front Microbiol. 2023 Jan 26;13:1076511. doi: 10.3389/fmicb.2022.1076511 (PMC9909597; doi:10.3389/fmicb.2022.1076511)
Supplement: Supplementary file 2 [file Image_1.pdf]

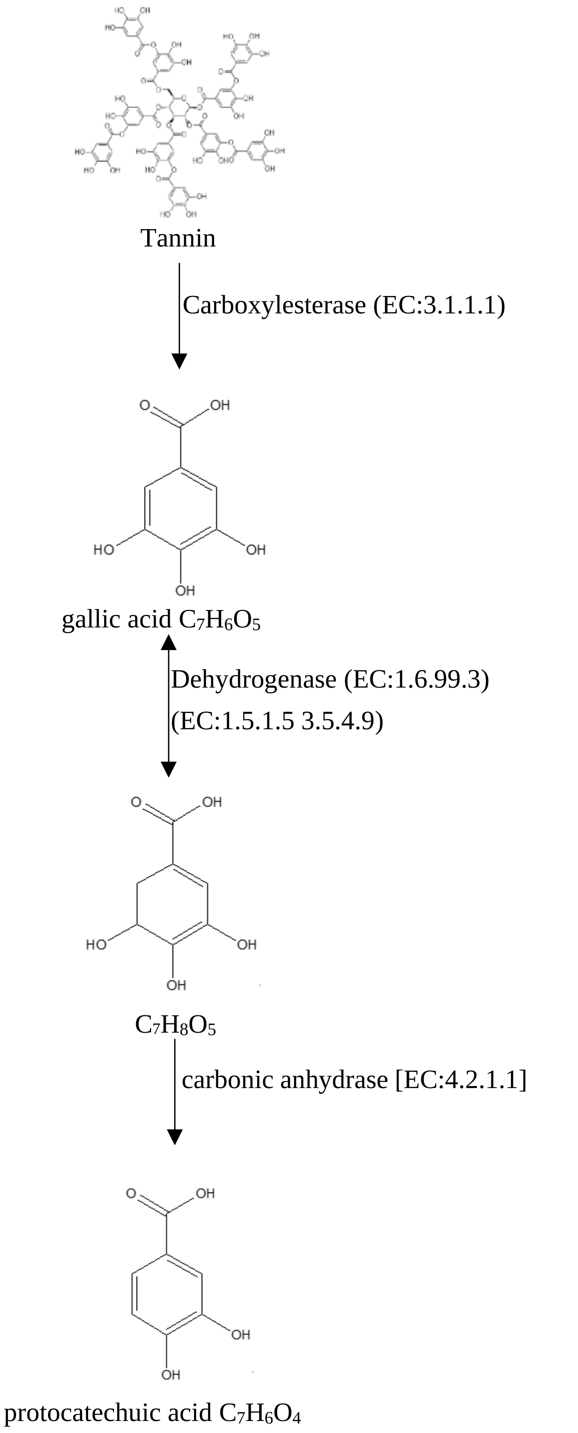

Fig. S1. Biosynthetic pathway of protocatechuic acid.

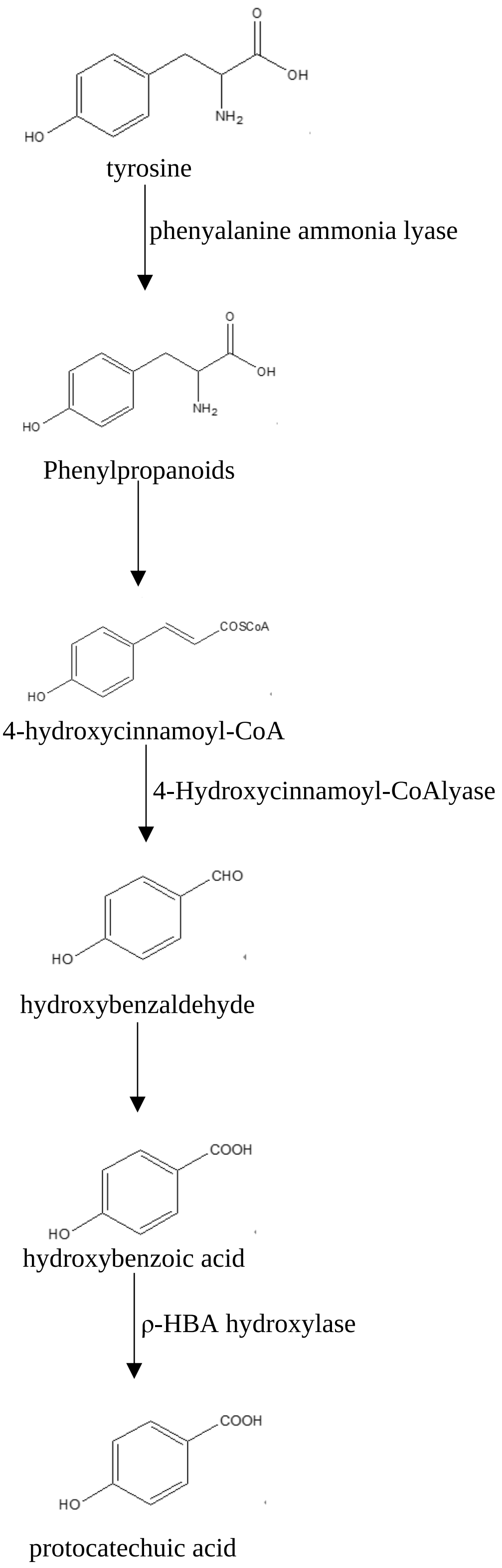

Fig. S2. Biosynthetic pathway of protocatechuic acid.

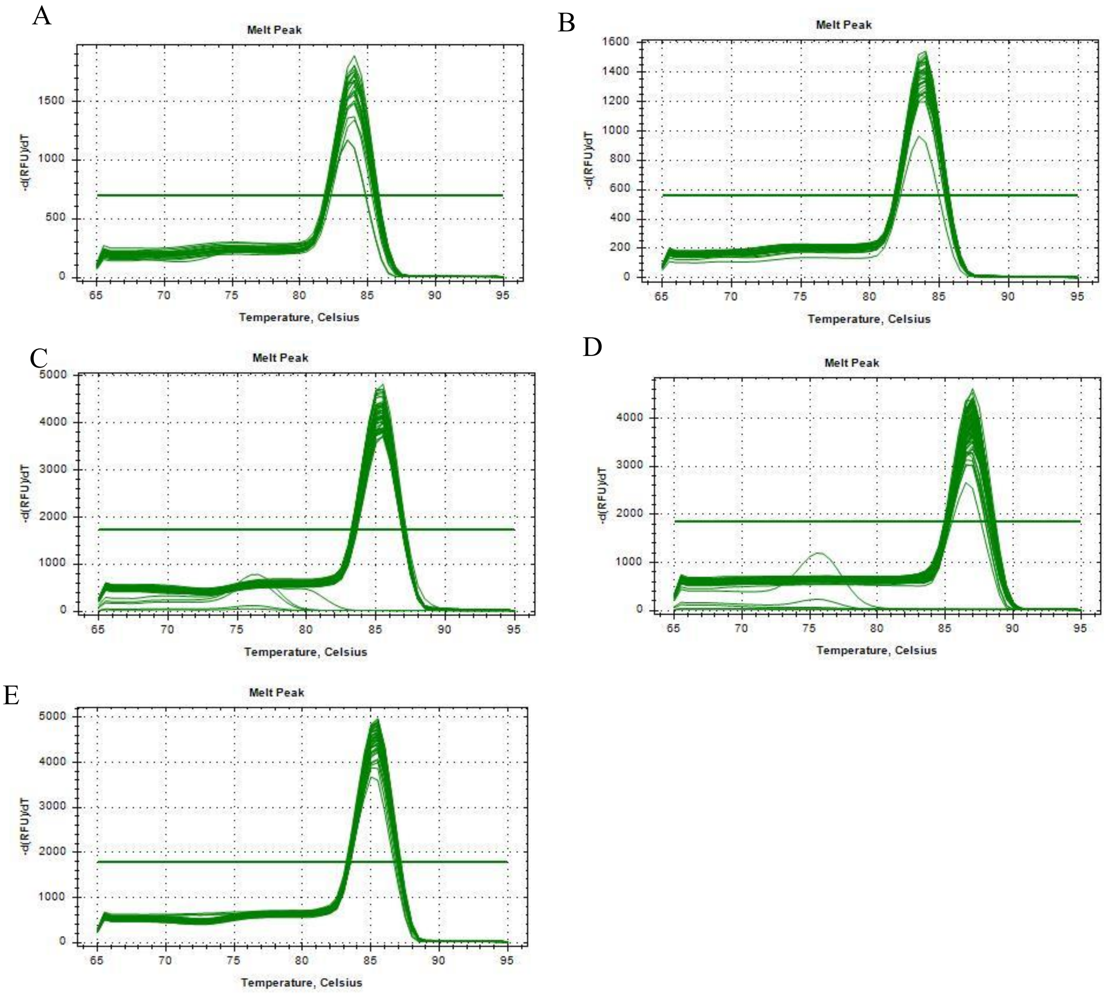

Fig. S3. The q-PCR real time melting curve of genes *patK, patN, patL, rds, rpt, prx1, prx2, prx3, prx4,* and *β-tubullin.* A: For *patK, patN* and *β-tubullin.* B For *patL* and *β-tubullin.* C For *rds, rpt* and *β-tubullin.* D For *prx1, prx2, prx3* and *β-tubullin.* E For *prx4* and *β-tubullin.* Data with non-standard solution curves were discarded.
